# Reducing catheter-associated urinary tract infections: a systematic review of barriers and facilitators and strategic behavioural analysis of interventions

**DOI:** 10.1186/s13012-020-01001-2

**Published:** 2020-07-06

**Authors:** Lou Atkins, Anna Sallis, Tim Chadborn, Karen Shaw, Annegret Schneider, Susan Hopkins, Amanda Bunten, Susan Michie, Fabiana Lorencatto

**Affiliations:** 1 Centre for Behaviour Change, University College London, Alexandra House, 7-19 Queens Square, London, WC1N 3AZ UK; 2 Public Health England, Wellington House, 133-155 Waterloo Road, London, SE1 8UG UK

**Keywords:** Catheter-associated urinary tract infection, CAUTI, Theory, Behaviour change wheel, Strategic behavioural analysis, Behaviour change techniques, Theoretical domains framework

## Abstract

**Background:**

Reducing the need for antibiotics is crucial in addressing the global threat of antimicrobial resistance. Catheter-associated urinary tract infection (CAUTI) is one of the most frequent device-related infections that may be amenable to prevention. Interventions implemented nationally in England target behaviours related to catheter insertion, maintenance and removal, but the extent to which they target barriers to and facilitators of these behaviours is unclear.

This strategic behavioural analysis applied behavioural science frameworks to (i) identify barriers to and facilitators of behaviours that lead to CAUTI (CAUTI-related behaviours) in primary, community and secondary care and nursing homes; (ii) describe the content of nationally adopted interventions; and (iii) assess the extent to which intervention content is theoretically congruent with barriers and facilitators.

**Methods:**

A mixed-methods, three-phased study: (1) systematic review of 25 studies to identify (i) behaviours relevant to CAUTI and (ii) barriers to and facilitators of CAUTI-related behaviours, classified using the COM-B model and Theoretical Domains Framework (TDF); (2) content analysis of nationally adopted CAUTI interventions in England identified through stakeholder consultation, classified using the Behaviour Change Wheel (BCW) and Behaviour Change Techniques Taxonomy (BCTTv1); and (3) findings from 1 and 2 were linked using matrices linking COM-B and TDF to BCW/BCTTv1 in order to signpost to intervention design and refinement.

**Results:**

The most frequently reported barriers to and facilitators of CAUTI-related behaviours related to ‘environmental context and resources’; ‘knowledge’; ‘beliefs about consequences’; ‘social influences’; ‘memory, attention and decision processes’; and ‘social professional role and identity.’

Eleven interventions aiming to reduce CAUTI were identifed. Interventions were primarily guidelines and included on average 2.3 intervention functions (1–5) and six BCTs (2–11), most frequently ‘education’, ‘training’ and ‘enablement.’ The most frequently used BCT was ‘information about health consequences’ which was used in almost all interventions. Social professional role and identity and environmental context and resources were targeted least frequently with potentially relevant BCTs.

**Conclusions:**

Interventions incorporated half the potentially relevant content to target identifed barriers to and facilitators of CAUTI-related behaviours. There were missed opportunities for intervention as most focus on shaping knowledge rather than addressing motivational, social and environmental influences. This study suggests that targeting motivational, social and environmental influences may lead to more effective intervention design and refinement.

Contributions to the literature

- Catheter-associated urinary tract infection is one of the most prevalent healthcare-associated infections, but it is unclear on the extent to which influences on CAUTI-related behaviours are targeted in current interventions.
- We found half the potentially relevant content to target identified barriers and facilitators in interventions but strategies to target motivational, social and environmental influences were largely missing in favour of those targeting knowledge.
- To our knowledge, this is the first attempt to apply theory-based, behavioural tools in the context of policy to identify influences on behaviour and assess the match between influences on behaviour and intervention content.

## Background

Improving infection prevention and control (IPC) is a crucial step in addressing the global health threat of antimicrobial resistance [1, 2]. Reducing the need for antibiotic use by preventing infections occurring requires behaviours to change in health care professionals (HCPs), patients and the general population across healthcare settings, e.g. primary, secondary and community care and nursing homes. Relevant HCP behaviours include keeping hands, equipment and environments clean, observing IPC protocols during invasive medical procedures (e.g. surgery, inserting catheters and other devices), continence care, and avoiding breach in skin or mucous membranes. Urinary tract infection (UTI) is one of the most common healthcare associated infections with approximately half associated with the presence of a urinary catheter [3]. These infections can be acquired by unnecessary use, poor insertion technique that can introduce bacteria, and leaving a catheter in too long allowing bacteria to travel up the catheter to the bladder causing UTI and potentially onward into the blood.

Catheter-associated urinary tract infections (CAUTI) are a product of a complex set of interrelated behaviours performed by multiple individuals. In the English National Health Service (NHS), interventions targeting behaviours that prevent CAUTI have been implemented at different levels from national evidence-based guidelines to local interventions to implement these guidelines. Some of these have been widely adopted across the country such as the Houdini protocol [4] (seven criteria for nurse-driven decision-making on catheter removal) and the catheter passport (patient-held record of catheter decision-making and care) [5].

It is not currently known on the extent to which barriers to and facilitators of behaviours related to CAUTI are targeted in current interventions. This is a notable gap considering interventions which target factors influencing behaviour (barriers and facilitators) are more likely to be effective in achieving behaviour change [6]. Investigating this can be facilitated by applying behavioural theory and evidence-based tools to determine the congruence (i.e. the match) between intervention content and barriers to and facilitators of CAUTI-related behaviours. Exploring theoretical congruence in the context of already widely implemented interventions enables strategic identification of opportunities for intervention and policy refinement. We termed this process ‘strategic behavioural analysis.’

**Fig. 1:**
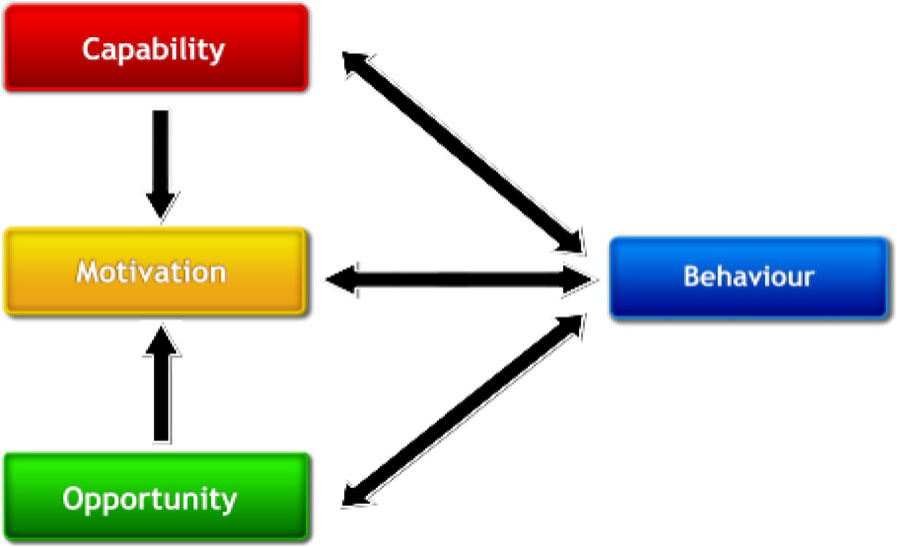
COM-B model

**Fig. 2:**
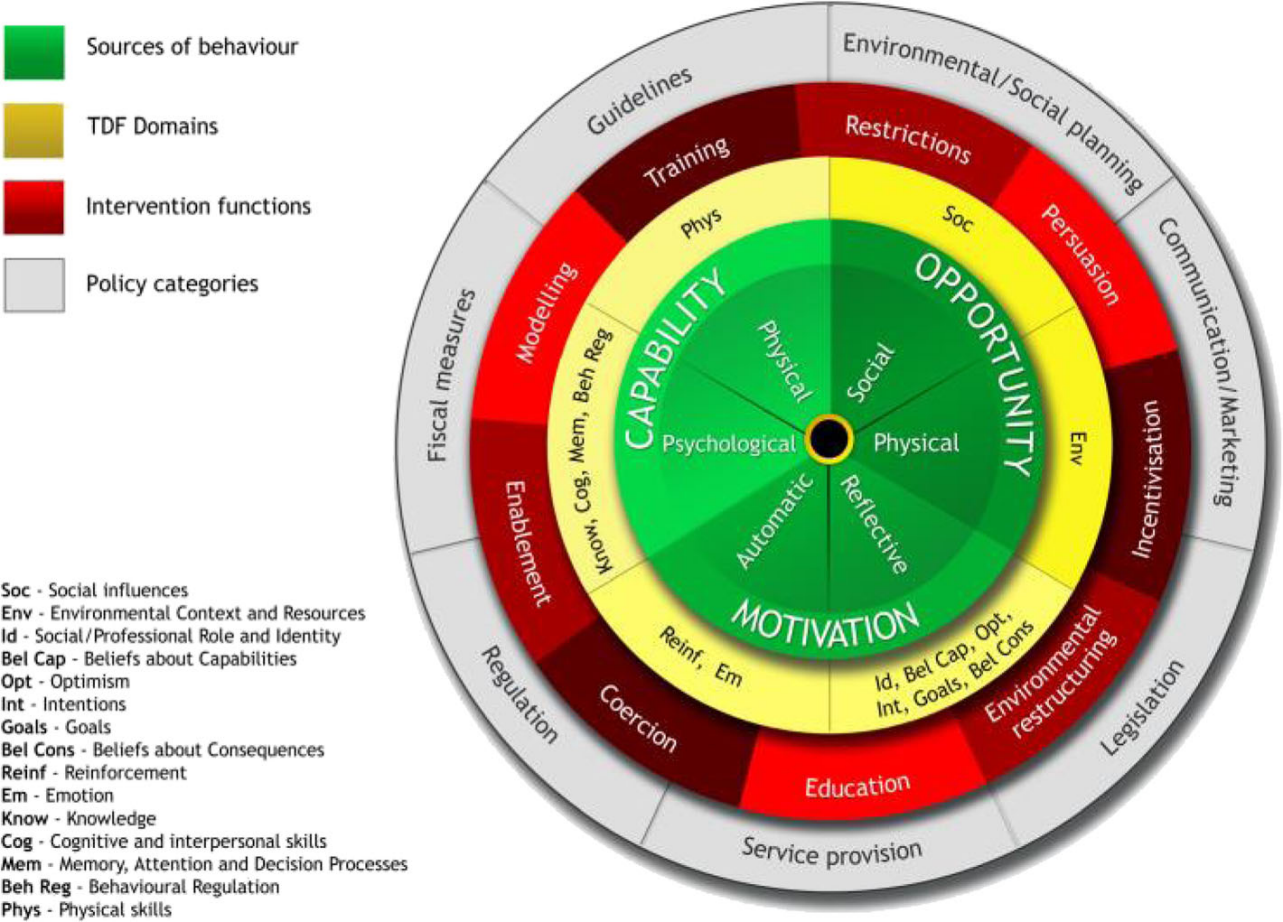
TDF domains linked to COM-B within the Behaviour Change Wheel

Tools such as the behaviour change wheel, which includes the theoretical model of behaviour COM-B (Fig. 1), the Theoretical Domains Framework (TDF), and the Behaviour Change Techniques Taxonomy (BCTTv1), may be specifically useful for describing intervention content and for investigating congruence between barriers to and facilitators of behaviour. Figure 2 shows how the TDF domains are linked to COM-B with the 14 more detailed domains and their associated constructs sitting within the broader COM-B model [see Additional file 1 for labels, definitions and examples for COM-B and TDF].

The behaviour change wheel (BCW), a synthesis of 19 frameworks of behaviour change, can be used to characterise interventions. COM-B sits at the ‘hub’ of the wheel and is surrounded by nine intervention functions, i.e. purposes an intervention serves and seven policy categories, i.e. channels through which interventions might be delivered (Fig. 2; see Additional file 2 for labels and definitions) [7]. Different intervention functions and policy categories are likely to be differentially effective depending on the extent to which capability, opportunity and/or motivation need to change [see Additional file 3 for matrices linking COM-B to intervention functions and policy categories].

How intervention functions are delivered can be described using a 93-item taxonomy of behaviour change techniques (Behaviour Change Techniques Taxonomy v1—BCTTv1) [8]. Links between intervention functions and BCTs (i.e. which BCTs might be considered to serve each intervention function) are described by Michie et al. 2014 [9].

Typically, these have been used to design new interventions. However, where there are already interventions in place that may not be working optimally, these tools have been applied to refine established interventions [10] and in guiding evidence synthesis of published evidence on interventions and barriers to and facilitators of behaviour. For example, the WIDeR-EyeS project (What Works to Increase Attendance for Diabetic Retinopathy Screening? An Evidence sYnthEsiS) conducted two reviews, one of interventions to increase attendance for diabetic retinopathy screening which were coded using BCTTv1 [11] and one of barriers to and facilitators of screening attendance, coded using TDF [12]. Findings from these reviews were compared using linking matrices to assess the extent to which BCTs in interventions targeted relevant barriers to and facilitators of the behaviours they were intended to change [13]. Using this method, the authors identified barriers to screening that were not targeted by the intervention BCTs, for example, emotional barriers.

This study had the following aims:

1. Identify barriers to and facilitators of CAUTI-related behaviours in HCPs
2. Describe the content of nationally adopted interventions in England to reduce CAUTI
3. Establish the extent to which barriers to and facilitators of CAUTI-related behaviours are targeted by nationally adopted interventions

## Methods

This mixed-methods study was conducted over three phases, each relating to one of the aims. Phase 1 was a systematic review of barriers to and facilitators of CAUTI-related behaviours, phase 2 was a content analysis of nationally adopted interventions in England to reduce CAUTI and phase 3 compared findings from phases 1 and 2 to establish the extent to which intervention content was theoretically congruent with the identified barriers to and facilitators of CAUTI-related behaviours.

### Aim 1—systematic review of barriers to and facilitators of CAUTI-related behaviours

#### Search strategy and selection criteria

We conducted a systematic review in accordance with PRISMA guidelines (Additional file 4). We searched MEDLINE, EMBASE and PsycINFO electronic databases in November 2017, limiting searches to 1995 onwards to optimise generalisability to current NHS settings and acknowledge the improvement in the quality of reporting in peer-review literature over time [see Additional file 5 for search strategies]. We included empirical qualitative and/or quantitative research or systematic review articles reporting barriers to and facilitators of CAUTI-related behaviours performed by HCPs in primary, secondary or community care or nursing homes, with titles and abstracts written in English.

#### Study selection and quality assessment

Titles and abstracts were screened against the inclusion and exclusion criteria independently by two reviewers. Full texts of studies meeting the inclusion and exclusion criteria were then screened against the same criteria by two reviewers. At both stages, any papers for which the decision was not clear were discussed with other members of the review group. We used the Mixed Methods Appraisal Tool (MMAT) [14] to assess the quality of qualitative, quantitative and mixed-methods studies.

#### Data extraction tools

Study characteristics extracted were country, setting, disease, participants, target behaviour and how the target behaviour was measured. Quotes and author interpretations of barriers and facilitators were coded using COM-B and TDF. Data were extracted by LA and checked by FL. Disagreements were resolved by discussion.

#### Data analysis

We conducted a three-step framework [15] and thematic [16] analysis to synthesise and explain barriers to and facilitators of CAUTI-related behaviours identified in the systematic review [13]:

1. Framework analysis by deductively coding extracted data according to barriers and facilitators into the COM-B and TDF domain(s) they were judged to represent best.
2. Thematic analysis within each TDF domain, grouping similar data points and inductively generating summary theme labels.
3. Ranking TDF domains in terms of importance according to frequency (number of studies), elaboration (number of themes) and evidence of conflicting beliefs within domains (e.g. if some participants report lack of knowledge of guidelines whereas others report familiarity with guidelines) [17]. Rank order was determined first by frequency, then elaboration, then evidence of conflicting beliefs.

These data are described using COM-B and TDF, with COM-B offering a higher-level summary and TDF offering a more granular level of analysis.

### Aim 2—content analysis of nationally adopted interventions in England to reduce CAUTI

#### Search strategy and selection criteria

We asked stakeholder delegates at IPC conferences, members of clinical commissioning groups, topic experts from relevant evidence-based guideline development groups and Public Health England Antimicrobial Resistance (AMR) Programme Board) to identify interventions aimed at HCPs to reduce CAUTI; > 100 responded.

#### Intervention selection and quality assessment

Interventions were limited to programmes, improvement strategies and policies currently adopted across England where the primary aim was to reduce CAUTI. Interventions that were implemented only locally were not included as the aim of this study was to understand interventions implemented at a national level.

#### Data extraction tools

The BCW and BCTTv1 were used to code content identified in materials or descriptions of interventions into intervention functions, policy categories and BCTs. Definitions provided in the BCW and BCTTv1 were referred to throughout the coding exercise to ensure coding was appropriate and consistent.

#### Data analysis

For each intervention, we recorded the total number of intervention functions, policy categories and BCTs and calculated the mean and range. We recorded the number of interventions in which each BCT and intervention function was present (mean and range), identified the most and least frequent intervention functions and BCTs and extracted representative examples across interventions.

### Aim 3—investigating congruence by linking identified barriers and facilitators to intervention content

BCTs are likely to be more or less effective depending on the nature of the barrier or facilitator. ‘Theoretical congruence’ is a term we use to define the extent to which BCTs in interventions are relevant to barriers and facilitators of behaviours the intervention is intended to change. BCTs can have different levels of congruence. For example, the BCT ‘behavioural rehearsal/ practice’ is likely to be relevant where there is a lack of capability to perform the behaviour. This would represent high congruence, i.e. a match between intervention content (BCT) and the barrier to the behaviour (lack of capability). In contrast, if lack of motivation was a barrier to the behaviour, then using the BCT ‘behavioural practice/rehearsal’ is unlikely to bring about behaviour change; this represents low congruence between intervention content and factors influencing behaviour [18].

Exploring congruence between intervention content and barriers to and facilitators of behaviour can be achieved using matrices which link both the techniques from BCTTv1 and BCW intervention functions to construct domains from both the TDF and COM-B, producing congruent BCT × domain pairings. These are based on expert consensus [13].

#### Data analysis

This analysis applied a matrix that included the BCT × TDF pairings published in Cane et al. [19] and Michie et al. [6] to investigate the level of theoretical congruence between the content of interventions targeting CAUTI and the published literature on factors influencing behaviours related to CAUTI [see Additional file 6 for matrix]. To achieve this, we took the following steps:

- Step 1. For each BCT identified in the existing interventions, we then consulted the TDF × BCT matrix to see which domains it was paired with. We then looked at findings from the systematic review to see whether the domain(s) the BCT was paired with was classified as a key domain. BCTs were then classified as having either:
  - Low congruence—did not target any key domains
  - Medium congruence—targeted at least one key domain
  - High congruence—targeted 2+ key domain s[13]
- Step 2. In addition to investigating the extent to which BCTs identified in interventions were linked to key domains (step 1), we also sought to establish, of the key domains we identified in the systematic review, which potentially relevant BCTs were suggested in the matrix. Some of the BCTs suggested by the matrix may have not been identified in existing interventions; these represent missed opportunities for intervention design. We examined the frequency with which these BCTs were identified in the existing interventions. We classified each potentially relevant BCT as follows:
  - Opportunity seized—instances where a theoretically congruent BCT (according to the matrix) was identified in an existing intervention at least once
  - Missed opportunity—instances where the theoretically congruent BCT was never identified in existing interventions
- Step 3. As there are multiple ways in which a single behaviour change technique can be delivered, we also examined whether BCTs we identified in interventions were delivered in a way that addressed the specific nature of the barriers and facilitators identified in key TDF domains. For example, if the BCT ‘information about health consequences’ was delivered in terms of providing information on the severity and complications of CAUTI, but a barrier within the domain ‘beliefs about consequences’ was the belief that inserting catheters increase convenience for the patient and healthcare professional, this would represent the lack of congruence between the content of the BCT and the identified barriers and facilitators within the corresponding domain; despite, *in theory*, this BCT being potentially relevant and congruent with this domain.
- We repeated steps 1–3 at the level of BCW intervention functions and policy categories, by consulting the matrices linking BCW to COM-B and TDF [9] to identify the extent to which intervention functions and policy categories in existing interventions target key COM-B and TDF domains influencing behaviour, and what additional intervention functions and policies may be of relevance to addressing barriers and facilitators within the most important domains.

## Results

### Systematic review of barriers of and facilitators to CAUTI-related behaviours

We identified 25 studies which met the inclusion criteria. Table 1 provides a summary of these studies. Seventeen were conducted in the USA with the remainder being conducted in France, Canada, UK, Australia, Taiwan and Thailand. The majority of studies (92%) were conducted in secondary care (including three in tertiary care). Only one study was conducted in nursing homes and one in community care; therefore, results are presented integrated across care settings [see Additional file 7 for a flow of information through the review].

**Table 1:** Summary of included studies

Reference	Country	Disease	Participants	Behaviour	Measurement of behaviour
Community care					
Getliffe & Newton [20]	UK	Not specified	District nurses (101/129 total sample; 18 community hospital and 10 nursing home care staff)	Record keeping relating to catheter care and CAUTI	Self-report questionnaire
Nursing home					
Krein et al. [21].	USA	Not specified	Organizational and facility leaders	Implementing ‘The Agency for Healthcare Research and Quality (AHRQ) Safety Program for Long-term Care: Health Care-Associated Infections/Catheter-Associated Urinary Tract Infection'	Semi-structured telephone interviews
Secondary care					
Krein et al. [22] Harrod et al. [23]	USA	Not specified	Infection control nurse (42), nurse/nurse manager (25), other, e.g. quality manager (2), hospital epidemiologist or infectious diseases physician (1); prevention specialists	Implementing the ‘Bladder Bundle’ care package	Semi-structured interview
Alexaitis & Broome [24]	USA	Neuroscience intensive care unit: common diagnoses include aneurysms, arteriovenous malformations, central nervous system neoplasms, traumatic brain injuries, spinal cord injuries, hemorrhagic and ischemic strokes, and status epilepticus.	Patients (183), nurses (107)	Discontinuation of indwelling catheters and use of bladder ultrasonography in conjunction with intermittent catheterizations	Pre-post study: catheter utilization, CAUTI rates, number of CAUTIs per month, LOS (length of stay, and cost associated with treating CAUTIs
Andreessen et al. [25]	USA	Not specified	Male in-patients with acute indwelling urinary catheters; staff of the medical centre	Implementing evidence-based guidelines and a urinary catheter bundle (Adult Catheter Bundle) focusing on optimizing the use of urinary catheters through continual assessment and prompt catheter removal.	Pre-post study: catheter device days, compliance with urinary catheter orders, and computer documentation of continued catheter indications.
Apisarnthanarak et al. [26]	Thailand	Not specified	Survey: general personnel; interview: lead infection preventionist	Prevention practices for CAUTI, CLABSI and VAP	Survey; interview assessing prevention practices
Bursle et al. [27]	Australia	Not specified	Patients with urinary source bloodstream infection associated with an indwelling urinary catheter	Insertion of urinary catheter.	Case-control study: assessing risk factors for urinary catheter associated bloodstream infection
Carter et al. [28] Carter et al. [29]	USA	Not specified	Staff at emergency department	Implementing a CAUTI prevention program among Emergency Departments	Qualitative comparative case study
Hu et al. [30]	Taiwan	Not specified	65 years or older	Insertion of urinary catheter	Prospective study: risk factors and outcomes for inappropriate use of urinary catheters
Conner et al. [31]	USA	Not specified	Nurses	Nurse driven early catheter discontinuation; assessing a patient’s need for indwelling urinary catheterization beyond 48 h	Pre-post study: factors associated with nurses’ adoption of an evidence-based practice to reduce the duration of catheterization
Conway et al. [32]	USA	Not specified	IPC (infection prevention control) department managers or directors	Adherence to CAUTI prevention policies	Cross-sectional survey on presence of CAUTI prevention policies, adherence to policies, CAUTI incidence rates
Crouzet et al. [33]	France	Not specified	Five hospital departments (not specified further)	Reducing the duration of the catheterisation	Non-random intervention study: duration of catheterisation, late CAUTI frequency
Dugyon-Escalante et al. [34]	USA	Not specified	Patients in intensive care units	Managing catheter use by multidisciplinary teams	Number of CAUTI cases and infection rates: pre-post
Fakih et al. [35]	USA	Not specified	Patients in medical-surgical units	Unnecessary use of urinary catheters	Quasi-experimental study with a control group: reduction in the rate of UC utilization
Fakih et al. [36]	USA	Not specified	Nurse and physician champions. Nurses caring for the patients. Other healthcare workers (e.g. infection preventionist, quality manager, safety officer, utilization manager)	Urinary catheter use and appropriateness of the indication for use (accountability at the unit level).	Symptomatic National Healthcare Safety Network (NHSN) CAUTI rate and population-based CAUTI rate. AHRQ's Hospital Survey on Patient Safety Culture administered both at baseline and 15 months later to evaluate changes in patient safety culture over time. Readiness assessment per unit at the beginning of the project and team check-up tool quarterly to report on progress with the implementation of CUSP principles and barriers
Gupta et al. [37]	USA	Not specified (ICU patients)	MICU medical director, MICU fellows, nurse managers and an infection control nurse	1. Restricting IUC use to a limited list of predetermined indications. 2. Physicians and nurses were required to discontinue urinary catheters in all patients on admission unless warranted. 3. Narrowing down the criteria for urinary catheter utilization to urinary retention and genitourinary procedures only. 4. Use of sonographic bladder scanning to identify high-risk patients who may need indwelling catheters in the near future	IUC utilization ratio (number of urinary catheter days/patient days) and catheter-associated urinary tract infection (CAUTI) rates (number of CAUTI infections in a particular location or number of urinary catheter days in a particular location × 1000)
Mann et al. [38]	Canada	Not specified (intensive care units and rehabilitation unit)	Intensive care and rehabilitation unit nurses	Compliance with CAUTI prevention measures (Foley maintenance)	Compliance with the following evidence-based practices: catheter securement, tamper evident seal (TES) intact, absence of dependent loop, catheter below bladder level, drainage bag not touching floor and drainage bag not overfilled
Murphy et al. [39]	UK	Not specified (ED, medical assessment unit, cardiology wards, and older people’s acute medicine wards)	8 nurses and 22 physicians in retrospective think aloud - RTA interviews. 20 of these (not specified how many nurses/physicians) also took part in a semi-structured interview	Decision making regarding IUC placement	30 RTA interviews and 20 semi-structured interviews
Patrizzi et al. [40]	USA	Not specified (ED and inpatient units)	ED nurses	Implementing a nurse-driven protocol to reduce CAUTI: Emergency department behaviours: 1. Removing direct access to catheters by placing them centrally in a supply closet instead of in each bedside supply cart. 2. Only storing 14F catheters (and no larger ones) in the supply closet as risk of infection increases with size. 3. Adding intermittent urinary catheterization kits to the supply closet as an alternative. 4. Education (e.g., The PPMC ‘UTI Bundle’ mandatory education day). 5. Availability of a bladder scanner. 6. New order set for indwelling urinary catheterization that lists 5 different indications to justify catheter placement (following hospital policy) instead of the previous ‘Foley catheter insertion’ order. 7. Collaboratively discussion between physician and nurse if the latter feels the insertion does not meet the established criteria. Inpatient unit behaviours: 1. Monitoring sheet placed on each patient’s medical record. 2. Daily assessment of a. necessity and b. standards for managing the catheter are being kept (e.g. bag below level of bladder)	Percentage of patients admitted from ER with indwelling urinary catheters
Smith L et al. [41]	USA	Not specified	Burn ICU nurses	Insertion, maintenance and removal of urinary catheters.	CAUTI rates and catheter utilization rates
Tertiary care					
Fakih et al. [42]	USA	Not specified	EPs and resident staff in ED	Adherence to guidelines for urinary catheter placement	Data on urinary catheter presence on emergency department arrival, placement of a urinary catheter in the emergency department, documentation of a physician order for urinary catheter placement, reasons for placement, and compliance with the indications were collected retrospectively reviewing the emergency department records
Trautner et al. [43]	USA	Not specified	169 physicians	Management of catheter-associated urine cultures	Self-report questionnaire
Kolonoski et al. [44]	USA	Not specified (post-acute units patients)	Physicians and nurses	Implementation of quality improvement programme to reduce CAUTI	Interview and point prevalence survey of Foley catheter use

Nine studies used quantitative descriptive designs, nine used qualitative designs, five used mixed methods and two used non-randomised designs. Study quality details are presented in Additional file 8.

**Fig. 3:**
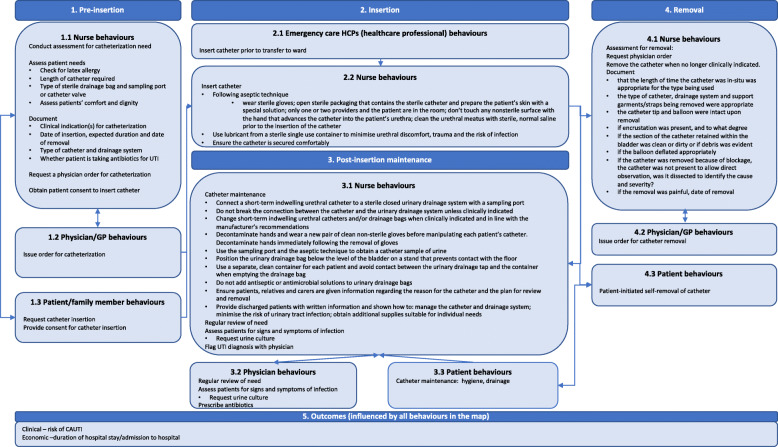
Map of CAUTI-related behaviours

Included studies typically focussed on barriers to and facilitators of implementing bundles of behaviours (i.e. multiple related behaviours) as part of interventions rather than individual behaviours (e.g. inserting a catheter, catheter removal, antibiotic prescribing) (Table 1). We summarised these behaviours according to four sequential time periods: pre-insertion of the catheter, insertion, post-insertion maintenance, and removal (see Fig. 3 for behavioural map).

Table 2 provides a ranking of the importance of COM-B and TDF domains identified. Additional file 9 presents the five most frequently identified themes within each COM-B and TDF domain. Table 3 indicates in which settings TDF domains were identified as barriers, facilitators or both. ‘Beliefs about capabilities’ was identified as a facilitator in secondary and tertiary care. ‘Behavioural regulation’ was identified as both barrier and facilitator in secondary care. All other identified TDF domains were classified as a mixture of either barrier or both barrier and facilitator across care settings. Additional file 10 contains a full list of all themes per domain, according to each care setting and corresponding CAUTI-related behaviour phase from the behavioural map (i.e. pre-insertion, insertion, post-insertion). The six most frequently identified domains are summarised below.

**Table 2:** Ranking of TDF domain importance according to the frequency of identification, thematic elaboration and evidence of conflicting beliefs

Ranking	TDF domain (COM-B)	Frequency (no. of studies identified in; max *n* = 25)	Elaboration (number of themes)	Evidence of barriers and/or facilitators within domains (Yes/No)
1	Environmental context and resources (physical opportunity)	13	8	Yes
2	Knowledge (psychological capability)	12	9	Yes
3	Beliefs about consequences (reflective motivation)	12	8	Yes
4	Social Influences (social opportunity)	9	6	Yes
5	Memory, attention and decision processes (psychological capability)	8	8	Yes
6	Social professional role and identity (reflective motivation)	6	4	Yes
7	Behavioural regulation	3	2	Yes
8	Beliefs about capabilities	2	2	No
Joint 9th and 10th	Skills	2	1	No
	Goals	2	1	No
Joint 11^th^ –14th	Reinforcement	0	0	–
	Intentions	0	0	–
	Optimism	0	0	–
	Emotions	0	0	–

**Table 3:**
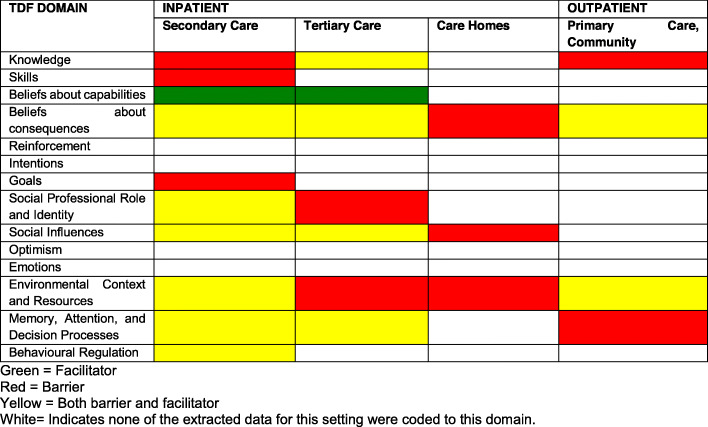
Classification of TDF domains as barriers, facilitators, or both across care settings

1. Environmental context and resources (*n* = 13 studies): Themes in this domain included ‘limited and inconsistent documentation and records relating to urinary catheter use’, i.e. absent or inappropriate documentation led to inappropriate catheter use; ‘transitions of care,’ e.g. when patients were moved between wards; ‘lack of time to perform alternatives to urinary catheterisation’ such as taking patients to the bathroom; ‘lack of available medical alternatives to urinary catheterisation’, e.g. a bladder scanner to determine catheter need (all of which were barriers to appropriate catheter usage) and ‘choice and availability of urinary catheters’ which was variable, i.e. available in some contexts and limited or absent in others, was coded as both a barrier and facilitator.

2. Knowledge (*n* = 12 studies): Barriers to appropriate catheter usage included lack of knowledge of ‘clinical guidelines; ‘duration of catheter insertion,’ i.e. not knowing that a catheter was in place or how long it had been in place; ‘risks associated with catheter use’; and ‘how to manage patients without catheterisation.’ ‘Knowledge of how to manage bacterial infections resulting from urinary catheterisation’ was classified as a facilitator of appropriate catheter usage.

3. Beliefs about consequences (*n* = 12 studies): Eight themes were identified. The most frequent was ‘convenience and ease of monitoring,’ e.g. inserting catheters for convenience purposes such as for measuring patients’ urine output or avoiding transfers to a bedpan or commode, which was identified in five studies. The theme ‘perceived severity of CAUTI’ was identified in two studies and classified as both a barrier and facilitator as some perceived CAUTI to be common and benign whilst others perceived it to be a potential source of risk for patients. ‘Lack of perceived benefits to interventions targeting CAUTI’ was identified as a barrier to appropriate catheter usage in two studies.

4. Social influences (*n* = 9 studies): Six themes related to this domain were identified. The most frequently identified were ‘requests from patients and their carers to have a catheter inserted’ (identified as a barrier in five studies); ‘lack of peer support and buy-in,’ i.e. low engagement amongst HCPs in performing CAUTI-related behaviours; ‘physicians dictating nurses’ practice,’ e.g. nurses complying with physician preference to leave a catheter in position and; and ‘cultural norms regarding standard catheterisation practice for specific patient groups,’ e.g. a standard practice of inserting a catheter for all patients in intensive care—these three themes were identified as barriers.

5. Memory, attention and decision making (*n* = 8 studies): Eight themes were identified in this domain including ‘pre-empting subsequent urinary catheterisation,’ i.e. inserting a catheter based on the perception the patient will anyway be catheterised in the future if not now (identified as a barrier in three studies); ‘catheterisation decisions based on non-medical criteria,’ e.g. to manage incontinence (identified as a barrier in one study); and ‘patient symptoms prompting investigation and treatment of possible CAUTI’ (identified as a facilitator in one study).

6. Social professional role and identity (*n* = 6 studies): Four themes related to this domain were identified including facilitators such as ‘having a hospital epidemiologist in post’ and ‘nurses leading change in urinary catheterisation practice’ and barriers such as ‘lack of acceptance of responsibility for urinary catheterisation decision making’ or ‘not perceiving CAUTI guidelines as relevant across hospital departments.’

### Content analysis of nationally adopted interventions in England to reduce CAUTI

We identified 11 interventions: six were implemented in primary care, seven in secondary care and eight in community care and in nursing homes (see Additional file 11 for intervention name and setting).

Additional file 12 shows the intervention functions, policy categories and BCTS identified in each intervention. Only two interventions targeted all behavioural phases: (i) The Health and Social Care Act 2008 Code of Practice on the prevention and control of infections and related guidance and (ii) Catheter Care: Royal College of Nursing Guidance for nurses. The majority focused on behaviours related to pre-insertion, insertion and post-insertion maintenance rather than removal.

### Intervention functions and policy categories identified in interventions

**Fig. 4:**
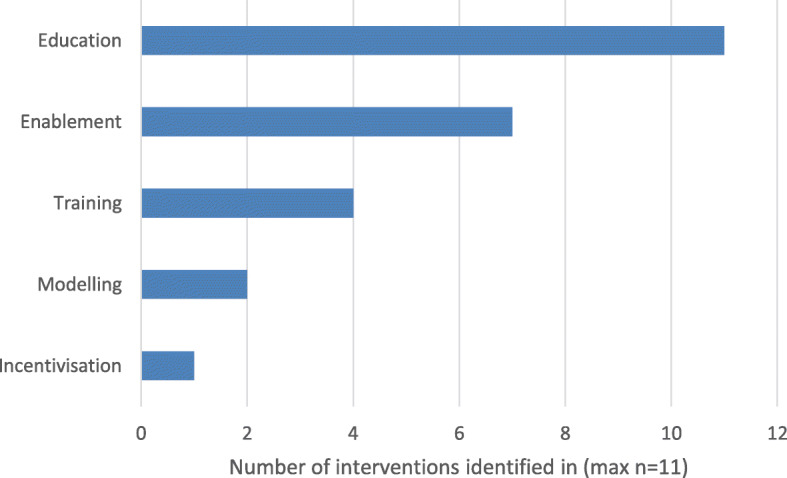
Frequency of identification of intervention functions

The mean number of intervention functions per intervention was 2·3 (1–5) (Fig. 4). All identified interventions served the function ‘education.’ Seven interventions served the function ‘enablement’ and four served the function ‘training.’ None of the interventions served the intervention functions ‘persuasion,’ ‘restructuring the environment,’ ‘restriction’ or ‘coercion.’

There was a very narrow range of policy strategies identified (mode = 1) (Additional file 12). The most frequently identified policy category was ‘guidelines’ (*n* = 9). One intervention, the ‘Health and Social Care Act 2008 Code of Practice on the prevention and control of infections and related guidance’ served the policy category ‘legislation.’ One intervention, the ‘Patient Safety Thermometer’ served the policy category ‘service provision.’

#### BCTs identified in interventions

**Fig. 5:**
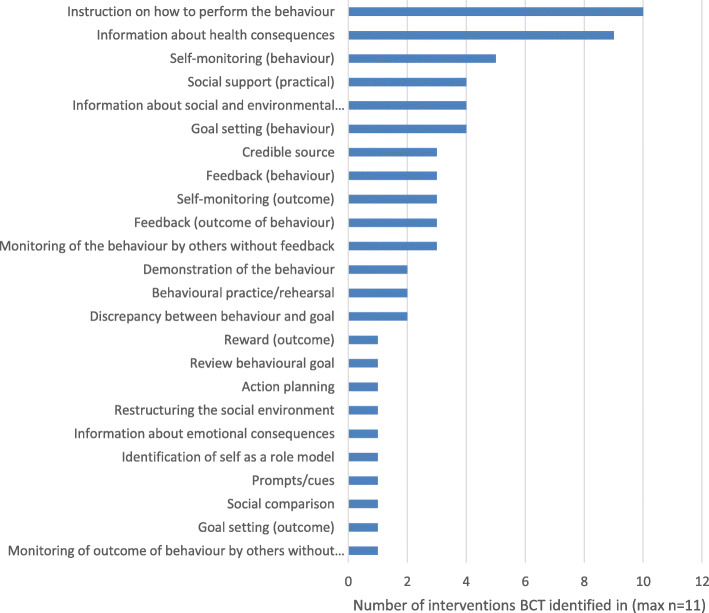
Frequency of identification of BCTs across interventions

The interventions typically included a narrow range of BCTs. The mean number of BCTs per intervention was 6 (2–11) (see Fig. 5). Many interventions also included ‘enablement’ functions, through ‘goal setting’, ‘monitoring’ and ‘feedback’ BCTs.

The BCT ‘instruction on how to perform the behaviour’ was identified in 10/11 interventions. Instruction was typically identified in guidelines, in the form of recommendations to perform the behaviour and how to do so, for a range of behaviours including those related to obtaining patient consent, catheter insertion, maintenance, removal and provision of patient information. The BCT ‘information about health consequences’ was identified in 9 interventions. Examples of how this BCT was delivered included, explaining that unnecessary treatment with antibiotics can increase the resistance of bacteria that cause urinary tract infections, making antibiotics less effective for future use.

Additional file 13 provides examples of how BCTs were operationalised in interventions

### Investigating congruence by linking identified barriers and facilitators to intervention content

The most frequently identified BCT, ‘instruction on how to perform the behaviour,’ was observed to have low theoretical congruence as the linking matrix suggests it is congruent with the TDF domain ‘skills,’ which was ranked a joint 9th out of 14 in terms of importance (Table 2). The second most frequent BCT, ‘information about health consequences,’ was observed to have high theoretical congruence as it was paired with two TDF domains rated as important—‘knowledge’ and ‘beliefs about consequences.’

Of the 24 BCTs identified in interventions, 10 BCTs had low theoretical congruence, six had medium congruence and nine had high theoretical congruence. BCTs with low congruence included those relating to ‘goal setting’ and ‘review’, ‘monitoring by others of behaviours or outcomes’, ‘instruction on how to perform the behaviour’, ‘behavioural practice/rehearsal’, ‘reward/outcome’ and ‘reframing’ meaning that the BCTs selected to deliver the intervention were not related to any of the TDF domains identified as important in systematic review data linked to barriers and facilitators. BCTs with high congruence related to ‘self-monitoring’ and ‘feedback’, ‘information about health and social and environmental consequences’ and ‘restructuring the social environment’ meaning that these BCTs would likely address the barriers or enable the facilitators to address the behaviours (see Table 4).

**Table 4:** Theoretical congruence between BCTs and TDF domains

BCT	Frequency ***(N*** interventions, Max 11)	Linked TDF domains according to integrated linking matrix^**a**^	TDF domain importance ranking^**b**^	Theoretical congruence between BCT and domain^**c**^
Feedback (on outcome of behaviour)	3	Knowledge	2	High
		Beliefs about consequences	3	
		Beliefs about capabilities	8	
		Goals	9–10	
Feedback (on behaviour)	3	Knowledge	2	High
		Beliefs about consequences	3	
		Beliefs about capabilities	8	
		Goals	9–10	
Self-monitoring (behaviour)	5	Memory, attention, decision processes	5	High
		Behavioural regulation	7	
		Skills	9	
		Beliefs about consequences	3	
		Beliefs about capabilities	8	
Self-monitoring (outcomes behaviour)	3	Memory, attention, decision processes	5	High
		Behavioural regulation	7	
		Skills	9	
		Beliefs about consequences	3	
		Beliefs about capabilities	8	
Social support (practical)	4	Social influences	4	High
		Beliefs about capabilities	8	
		Social professional role and identity	6	
		Intentions	11–14	
		Goals	9–10	
Information about health consequences	9	Knowledge	2	High
		Beliefs about consequences	3	
Information about social environmental consequences	4	Knowledge	2	High
		Beliefs about consequences	3	
Prompts/cues	1	Memory, attention, decision processes	5	High
		Environmental context and resources	1	
		Behavioural regulation	7	
Restructuring the social environment	1	Social influences	6	High
		Environmental context and resources	1	
Action planning	1	Goals	9–10	Med
		Intentions	11–14	
		Memory, attention, decision processes	5	
		Behavioural regulation	7	
Information about emotional consequences	1	Knowledge	2	Med
		Emotions	11–14	
Social comparison	1	Social influences	4	Med
Demonstration of the behaviour	2	Social influences	4	Med
		Skills	9	
Credible source	3	Beliefs about consequences	3	Med
		Goals	9–10	
		Intentions	11–14	
Identification of self as a role model	1	Social influences	4	Med
Goal-setting (behaviour)	4	Behavioural regulation	7	Low
		Skills	9	
		Beliefs about capabilities	8	
		Goals	9–10	
		Intentions	11–14	
Goal-setting (outcome)	1	Behavioural regulation	7	Low
		Skills		
		Beliefs about capabilities	8	
		Goals	9–10	
		Intentions	11–14	
Review behaviour goal(s)	1	Goals	9–10	Low
		Intentions	11–14	
Discrepancy between current behaviour and goal(s)	2	None	n/a	Low
Monitoring of outcome of behaviour by others without feedback	1	Skills	9	Low
Monitoring of the behaviour by others without feedback	3	Skills	9	Low
Instruction on how to perform the behaviour	10	None	N/A	Low
Reward (outcome)	1	Skills	9	Low
		Reinforcement	11–14	
		Goals	9–10	
		Intentions	11–14	
Behavioural practice/rehearsal	2	Skills	9	Low
		Beliefs about capabilities	8	

^a^Merged matrix combing Cane et al. [19] and Michie et al. [6] TDF x BCT linking matrices

^b^Domain ranking based on thematic analysis of barrier/facilitator literature (see Table 2)

^c^Classification of theoretical congruence: **Low:** BCT is not paired with any of the 6 key domains identified as important in the thematic analysis; **Medium:** BCT is paired with at least one domain identified as important; **High:** BCT is paired with two or more domains identified as important

#### Missed opportunities for intervention design and refinement

Table 5 shows whether intervention functions identified in the 11 interventions appropriately targeted the six most important TDF domains. According the matrix TDF domains ‘knowledge’ and ‘memory, attention, decision-processes’ could potentially be targeted by the intervention functions ‘education’, ‘training’ and ‘enablement.’ These intervention functions were identified in 11, four and seven interventions, respectively.

**Table 5:**
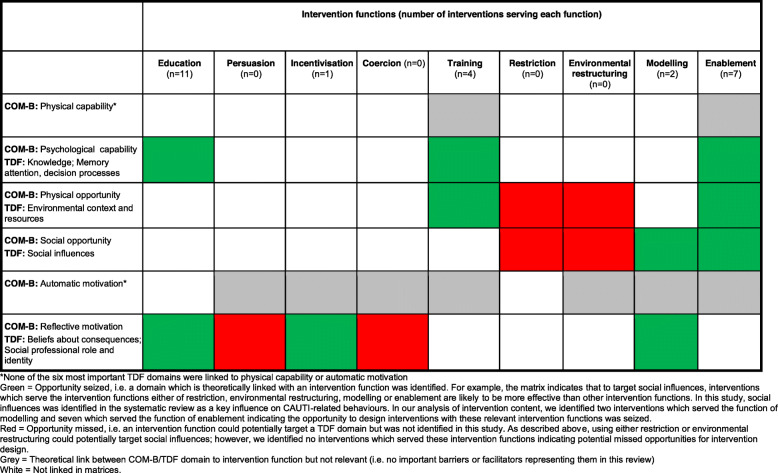
Seized and missed opportunities: intervention functions

Barriers and facilitators related to the TDF domain ‘environmental context and resources’ could potentially be targeted by the following intervention functions: ‘training’, identified in four interventions; ‘enablement’, identified in seven interventions; and ‘restriction’ and ‘environmental restructuring’ which were not identified in any interventions representing missed opportunities to target these barriers and facilitators.

Barriers and facilitators related to the TDF domain ‘social influences’ could potentially be targeted through the intervention functions ‘modelling’ and ‘enablement’ (identified in two and seven interventions, respectively). They could also be targeted through the intervention functions ‘restriction’ and ‘environmental restructuring’ which were not identified in any interventions again representing missed opportunities to target these barriers and facilitators.

The TDF domains ‘beliefs about consequences’ and ‘social professional role and identity’ could potentially be targeted through the intervention functions ‘education’, ‘persuasion’, ‘incentivisation’, ‘coercion’ and ‘modelling.’ ‘Education’ was identified in all interventions. ‘Coercion’ and ‘persuasion’ were not identified in any interventions representing a missed opportunity in all interventions. ‘Incentivisation’ was identified in one intervention and ‘modelling’ in two interventions. Whilst these are theoretically appropriate, they were identified in only a few interventions indicating that the majority of interventions missed opportunities to target barriers and facilitators related to ‘beliefs about consequences’ and ‘social professional role and identity’ using these domains.

Table 6 shows whether intervention functions identified in the 11 interventions were delivered through policy categories suggested by the BCW intervention function × policy category matrix. All intervention functions were delivered through at least one policy category suggested by the matrix. There was one instance in Catheter Care: Royal College of Nursing Guidance for nurses where the intervention function ‘modelling’ was delivered through ‘guidelines’ (observe catheterisation performed by others on actual patients) which is not suggested by the matrix. This function was also delivered through the appropriate policy category of ‘service provision’ and there was a missed opportunity for it to be delivered through ‘communication and marketing.’

**Table 6:**
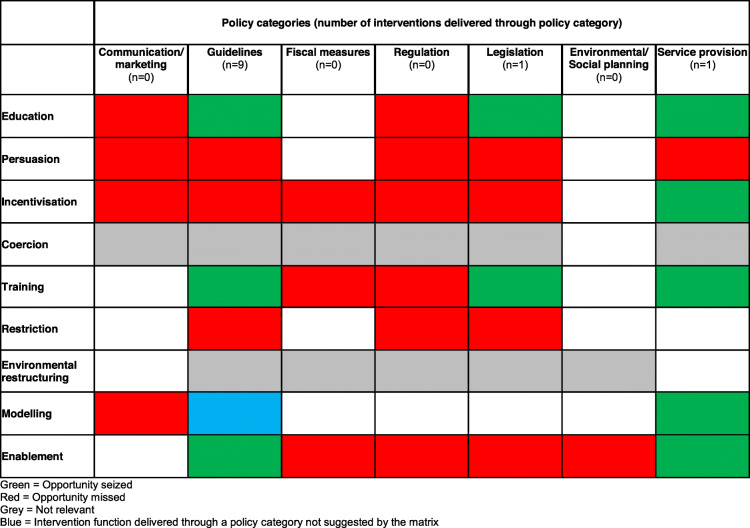
Seized and missed opportunities: policy categories

There were missed opportunities to deliver all intervention functions identified in interventions through the policy category of ‘regulation.’ ‘Communication/marketing’, ‘fiscal measures’ and ‘environmental and social planning’ are also three potentially relevant policy categories to support identified intervention functions but were never utilised

In terms of the extent to which BCTs targeted the TDF domains in which the majority of barriers to and facilitators of CAUTI-related behaviours were identified: nine BCTs targeted two or more of the six most important TDF domains (see Table 2) and were thus classified as having high theoretical congruence, i.e. a ‘match’ between the intervention content and key barriers to and facilitators of CAUTI-related behaviours. These high congruence BCTs were ‘feedback (on outcome of behaviour)’; ‘feedback (on behaviour)’; ‘self-monitoring (behaviour)’; ‘self-monitoring (outcomes behaviour)’; ‘social support (practical)’; ‘information about health consequences’; ‘information about social environmental consequences’; ‘prompts/cues’; and ‘restructuring the social environment.’ Six further BCTs were classified as having medium theoretical congruence, in that they targeted only one of the six most important TDF domains: ‘action planning’, ‘information about emotional consequences’, ‘social comparison’, ‘demonstration of the behaviour’, ‘credible source’, and ‘identification of self as a role model.’ However, nine BCTs in currently implemented interventions were classified as having low theoretical congruence, in that they do not target any of the six most important TDF domains influencing CAUTI-related behaviours: ‘goal-setting (behaviour)’. ‘goal-setting (outcome)’, ‘review behaviour goal(s)’, ‘discrepancy between current behaviour and goal(s)’, ‘monitoring of outcome of behaviour by others without feedback’, ‘monitoring of the behaviour by others without feedback’, ‘instruction on how to perform the behaviour’, and ‘reward (outcome)’.

Additional file 14 presents the frequency with which BCTs paired with important TDF domains were identified in existing interventions. BCTs paired with five of the six most important TDF domains (‘knowledge’, ‘beliefs about consequences,’ ‘social influences,’ ‘social professional role and identity’ and ‘environmental context and resources) were not used frequently (i.e. less than 60%) in existing interventions. This indicates numerous missed opportunities for intervention design. Opportunity seized was highest for the TDF domains ‘memory, attention and decision processes’ (100% of the theoretically coherent BCTs were used at least once in interventions) and ‘knowledge’ (57% of the theoretically coherent BCTs were used at least once in interventions). The most missed opportunities were for the TDF domains social professional role and identity and environmental context and resources.

Based on the investigation of the fit between identified barriers and facilitators and BCTs, there are numerous opportunities for further intervention design and refinement. Table 7 provides initial recommendations of potential strategies to address the more frequently identified (*n* > 3 studies) barriers and facilitators within the six most important TDF domains. These recommendations include examples of different ways of delivering BCTs that are already included in existing interventions, or additional, new BCTs that have not been identified in current interventions but are relevant to the key TDF domains [see Additional file 6]. The hypothetical example deliveries of these BCTs are intended as illustrations for how they might be operationalised; however, in moving forward with this work, the delivery of these BCTs should be co-designed with stakeholders with contextual understanding of the subject area using explicit criteria.

**Table 7:** Recommendations for intervention design and refinement

Theme	Proposed new BCT	Example delivery to address theme
Environmental context and resources		
Limited and inconsistent documentation and records	Restructuring the physical environment; prompts/cues	Creating standardised computer-based documentation requiring staff to enter reason for catheterisation, date of insertion, etc. (i.e. not circumvent system by leaving fields blank).
Transitions of care	Restructuring the social environment	Creating the rule that ward staff transferring patients to another ward check with the staff receiving the patient whether catheterisation is necessary (this rule could be prompted by a checklist for transfer of patients to another ward/hospital or home where staff check if the catheter is needed).
Lack of time to perform alternatives to urinary catheterisation	Adding objects to the environment	Provision of condom catheters, female urination devices and/or local commodes at bedside.
Knowledge		
Lack of knowledge of clinical guidelines and local procedural documents	Information to consider including in guidelines/local procedural documents: • Alternatives to catheterisation • How to safely manage infections arising from catheterisation? Whilst the information contained in the guidelines appears to address lack of knowledge in, e.g. link between catheter duration and CAUTI, the issue may be more to do with dissemination. Guideline implementation strategies to accompany recommendations may promote this.	
Beliefs about consequences		
Convenience and ease of monitoring	Anticipated regret and/or salience of consequences	Getting staff to think about how they would feel if a patient was diagnosed with CAUTI after they had catheterised them for non-medical reasons (this could be delivered as part of a training programme, staff meetings, printed and electronic materials).
	Pros and Cons	Encouraging staff to list the benefits and disadvantages of catheterising for convenience compared with catheterising for medical reasons (this could be delivered as a part of a training programme or suggested face to face in staff).
	Salience of consequences	Providing images emphasising the severity of CAUTI.
	Persuasive communication (Credible source)	Members of Trust leadership and senior members of staff endorsing not catheterising for convenience.
Social influences		
Requests from patients and their carers	Social comparison	Staff convey to patients/carers that most patients/carers do not request catheters and explain the reason why this is.
	Demonstration of the behaviour	Staff role modelling challenging patient/carer requests.
Lack of peer support and buy-in	Information about others’ approval	Informing staff engagement with CAUTI-reducing practices is encouraged by peers/senior staff.
Physicians dictate nurses’ practice	Restructuring the social environment	Strategies to empower nurses to lead on catheter decision-making (delivered through peers/senior team members).
	Social comparison	Provide examples of where the HOUDINI protocol has been effectively implemented.
Cultural norms regarding standard catheterisation practice for specific patient groups	Social comparison	Compare rates of catheterisation and corresponding rates of infection between wards/hospitals/primary care practices/nursing homes. Stratifying by professional role will increase the salience of this comparison.
Memory attention and decision processes		
Pre-empting subsequent urinary catheterisation	Action planning	Plan who will assess the patient for catheterisation and where this will happen
	Self-monitoring of behaviour	Document the action plan (see above) so there is agreement between staff on different wards whether the patient being transferred requires a catheter and if so, who will insert the catheter.

Social professional role and identity is not included as no theme was identified in 3 or more studies

Whilst the BCTs suggested here are linked to multiple intervention functions—the matrix in Table 5 indicates the most relevant functions interventions need to serve are ‘restructuring the social and physical environment’ (none of the identified interventions serve this function), ‘persuasion’ and ‘enablement.’

## Discussion

### Summary of findings

The aims of this study were to identify barriers to and facilitators of CAUTI prevention behaviours, describe the content of nationally adopted interventions and assess the extent to which intervention content is theoretically congruent with facilitators and barriers. Interventions incorporated half the potentially relevant content to target identifed barriers to and facilitators of CAUTI-related behaviours. There were missed opportunities for intervention as most focus on shaping knowledge rather than addressing motivational, social and environmental influences.

The most frequently identified barriers and facilitators related to the TDF domains: (1) ‘environmental context and resources’, e.g. having the time and equipment to perform alternatives to catheterisation; (2) ‘knowledge’. e.g. lack of knowledge of relevant clinical guidelines; (3) ‘beliefs about consequences’, e.g. healthcare professionals’ perceptions of severity of CAUTI and of the ease and convenience associated with catheterisation; (4) ‘social Influences’, e.g. family requests to catheterise the patient; (5) ‘memory, attention and decision processes’, e.g. making catheter-related decisions based on non-medical criteria; and (6) ‘social professional role and identity’, e.g. accepting responsibility for making catheter-related decisions.

We identified 11 interventions to reduce CAUTI that are implemented currently in England. These were typically in the form of clinical guidelines. All 11 interventions served the function ‘education’, seven served an ‘enabling’ function and four served a ‘training’ function. We identified 24 behaviour change techniques (BCTs) across the interventions. The most frequently identified BCTs were ‘instruction on how to perform a behaviour’ (identified in 10 interventions) with low theoretical congruence and ‘information about health consequences’ (identified in 9 interventions) which had high theoretical congruence. The BCT ‘self-monitoring of behaviour’ was identified in five interventions.

We took a generous and inclusive approach to our coding in that many interventions were *prompting* techniques such as monitoring, feedback and planning, rather than providing these techniques directly (e.g. guidelines including recommendations to monitor and feedback on CAUTI-related practice). We still coded for the presence of the techniques in such instances.

Combined, these findings highlight that interventions were primarily educational in nature whereas many of the barriers concerned the social and environmental context, and motivational influences of beliefs about consequences and perceptions of role. This suggests many missed opportunities for potentially effective interventions.

A number of ‘missed opportunities,’ for intervention design were identified in the form of theoretically congruent BCTs that are not currently included in interventions. This was particularly apparent for the TDF domains ‘environmental context and resources,’ and ‘social professional role and identity,’ for which only approximately a third of theoretically congruent BCTs were included (at least once) in existing interventions. For some TDF domains, such as ‘memory, attention and decision processes,’ a large proportion of congruent BCTs featured in existing interventions. These were delivered at a very low frequency suggesting a further missed opportunity for intervention design and refinement.

### Strengths and limitations

There are five limitations to this study. First, bundles rather than specific behaviours tended to be the focus of studies included in the systematic review. Therefore, it was not possible to identify which barriers and facilitators related to which specific behaviours and, in turn, to provide more specific recommendations for intervention development.

Secondly, as this is a secondary content analysis of published (including grey) literature, intervention descriptions are often poorly and vaguely specified and offer limited detail for coding. In addition, we were only able to synthesise barriers and facilitators that were reported by the authors of the included studies, raising the possibility of reporting/interpretation bias. As none of the included studies investigated barriers and facilitators using the TDF, it is possible that some of the TDF domains were not frequently identified because questions were not asked to assess the role that domain plays in influencing CAUTI-related behaviours; for example, we might hypothesise that reinforcement and emotion are likely to be important.

Thirdly, the majority of studies reporting barriers to and facilitators of CAUTI behaviours were conducted in the USA. The different geographical and health care contexts between the USA and England may limit the validity of this generalisation. Whilst the barriers and facilitators identified in the two UK studies identified in this review are comparable with non-UK studies in this review, widening the scope to the international literature allowed us to draw more robust conclusions on the barriers to and facilitators of CAUTI-related behaviours. Optimising the literature in this way has been done previously. For example, Lawrenson et al.’s 2018 HTA assessment of diabetic retinopathy screening was conducted primarily in the context of the UK NHS Diabetic Eye Screening Programme but included mainly studies conducted in the USA [11].

Fourthly, linking identified barriers and facilitators to CAUTI prevention behaviours with intervention content was done at an aggregate not individual level to signpost to seized and missed opportunities for intervention. Further work is required to establish the specific implications of these opportunities for individual-level interventions.

Finally, the TDF domain × BCT pairings upon which the assessments of congruence are discussed are the result of expert consensus, rating whether a TDF domain could be linked to a BCT in general (i.e. no specific context) and so do not differentiate between theoretically congruent pairings according to different types of behaviours. Therefore, some of the proposed theoretically congruent BCTs may not be as relevant or appropriate in the context of CAUTI.

An important factor to consider in interpreting these data is the frequency with which BCTs were identified. For example, whilst BCTs ‘feedback on the outcome of behaviour’ and ‘information about health consequences’ were both classified as having high theoretical congruence, ‘feedback on the outcome of behaviour’ was identified in only three interventions, whereas ‘instruction on how to perform the behaviour’ which had low theoretical congruence was identified in 10 interventions. We were inclusive in this linking element and so these findings may present an optimistic scenario and the actual degree of congruence could be lower than might seem.

### Future research directions

The factors influencing CAUTI-related behaviours are likely to vary and differ across HCPs and settings. Further research is needed to prioritise the key behaviours influencing CAUTI within complex intervention bundles. This would allow a more focused behavioural analysis to identify the barriers to and facilitators of CAUTI-related behaviours. Using a comprehensive behavioural theory or framework, such as the TDF or COM-B, will ensure that the wide range of potential barriers to and facilitators of behaviour are considered—from individual-level factors to broader social and physical environmental factors. This will build on the findings here as to support establishing whether TDF domains not identified in this systematic literature are relevant to CAUTI prevention behaviours.

Most studies included in our analysis investigated the barriers to and facilitators of CAUTI-related behaviours in secondary care. However, the majority of the existing interventions analysed target primary care, community and nursing homes. This is an important discrepancy and limitation to the linking exercise we conducted to establish whether existing interventions target the key factors influencing CAUTI-related behaviours. The factors influencing CAUTI-related behaviour are likely to be context specific and thus differ across care settings. Therefore, further behavioural analysis and diagnosis research in under-investigated care settings would enable a more accurate linking of existing interventions to influences on behaviour and more targeted recommendations for intervention development.

This descriptive review did not investigate which BCTs were associated with improved outcomes in existing interventions. Subsequent work could include reviews of the published, peer-reviewed evaluations of interventions targeting CAUTI, coupled with BCT coding of these interventions and meta-regression to identify which BCTs, intervention functions and policy categories are linked to effective interventions.

Given a key finding in this work was that there was a lack of awareness of guidelines, more process evaluation research is recommended to identify why guidelines are not being implemented. Current strategies tend to be ‘passive’ in that they are published in the public domain, but there is no clear active dissemination to those for whom the guidelines are most relevant. Investment in the development and evaluation of implementation intervention strategies would be a first step in reducing the evidence-implementation gap.

## Conclusions

The interventions identified in this work used a narrow range of strategies—primarily educational and often delivered in the form of guidelines. To better address barriers and facilitators identified in the systematic review, more proactive strategies are needed to increase the implementation of these guidelines [45]. Strategies could include effective communication to target audiences when guidelines are published across all settings; clear summary documents with key messages; implementation plans to facilitate the translation of recommendations into practice; supporting materials, e.g. training slides; auditing hospitals, GP practices and care homes against recommendations in guidelines and providing feedback on performance against these recommendations; highlighting discrepancies between observed and desired behaviours; and setting goals and action plans to reduce any observed discrepancy. Strategies such as these could also incorporate elements of social comparison such as comparing performance against other wards, teams or hospitals.

To our knowledge, this is the first time the combined behavioural tools of BCW, TDF and BCTTv1 have been applied in a policy context to understand the factors influencing a behaviour, characterise existing interventions and establish the congruence (i.e. match) between influences on behaviour and intervention content. These findings signpost policy-makers to where opportunities have been realised and missed in existing interventions to inform intervention refinement and the design of new interventions. In this and the WIDeR-EyeS study [13], peer-review evidence was synthesised and triangulated over 11 interventions. This method could also be applied to novel data on barriers and facilitators (e.g. interviews with relevant stakeholders) and triangulated with an individual intervention. At whichever level they are applied, these methods could increase understanding across health protection, public health and other areas of implementation research.

## Supplementary information

**Additional file 1.** Labels, definitions and examples of COM-B and Theoretical Domains Framework

**Additional file 2.** Behaviour Change Wheel labels, definitions and examples

**Additional file 3.** BCW matrices

**Additional file 4.** Adherence to reporting guidelines

**Additional file 5.** Electronic search strategies

**Additional file 6.** Merged Theoretical Domain Framework x Behaviour Change Techniques matrices

**Additional file 7.** Flow of information through the systematic review

**Additional file 8.** MMAT study quality rating

**Additional file 9.** Summary of barriers to and facilitators of CAUTI-related behaviours

**Additional file 10.** All reported barriers and facilitators within each TDF domain** identified across all care settings (primary, community, secondary and tertiary care, and care homes), nested according to COM-B

**Additional file 11.** Description of included nationally adopted interventions in England to reduce CAUTI

**Additional file 12.** BCTs, intervention functions and policy categories identified in each intervention

**Additional file 13.** Examples of BCT identification

**Additional file 14.** Opportunities for intervention design: the frequency with which theoretically congruent BCTs with important theoretical domains were used in existing interventions

## Data Availability

The datasets used and/or analysed during the current study are available from the corresponding author on reasonable request.

## References

[1] Domin MA (1998). Highly virulent pathogens--a post antibiotic era?. Br J Theatr Nurs.

[2] Fishman N (2006). Antimicrobial stewardship. Am J Med.

[3] Loveday HP, Wilson JA, Pratt RJ, Golsorkhi M, Tingle A, Bak A (2014). epic3: national evidence-based guidelines for preventing healthcare-associated infections in NHS hospitals in England. J Hosp Infect.

[4] Adams D, Bucior H, Day G, Rimmer J-A (2012). J Infect Prev.

[5] Codd J (2014). Implementation of a patient-held urinary catheter passport to improve catheter management, by prompting for early removal and enhancing patient compliance. J Infect Prev.

[6] Michie S, Johnston M, Francis J, Hardeman W, Eccles MP (2008). Appl Psychol.

[7] Michie S, van Stralen MM, West R (2011). The behaviour change wheel: a new method for characterising and designing behaviour change interventions. Implement Sci.

[8] Michie S, Richardson M, Johnston M, Abraham C, Francis J, Hardeman W (2013). Ann Behav Med.

[9] Michie S, Atkins L, West R (2014). The behaviour change wheel - a guide to designing interventions.

[10] Steinmo SH, Michie S, Fuller C, Stanley S, Stapleton C, Stone SP. Bridging the gap between pragmatic intervention design and theory: using behavioural science tools to modify an existing quality improvement programme to implement “sepsis six”, Implementation science. 2016;11(1):14. 10.1186/s13012-016-0376-8 PMC4739425 26841877

[11] Lawrenson JG, Graham-Rowe E, Lorencatto F, Burr J, Bunce C, Francis JJ (2018). Interventions to increase attendance for diabetic retinopathy screening. Cochrane Database Syst Rev.

[12] Graham-Rowe E, Lorencatto F, Lawrenson JG, Burr JM, Grimshaw JM, Ivers NM (2018). Barriers to and enablers of diabetic retinopathy screening attendance: a systematic review of published and grey literature. Diabet Med.

[13] Lawrenson JG, Graham-Rowe E, Lorencatto F, Rice S, Bunce C, Francis JJ (2018). What works to increase attendance for diabetic retinopathy screening? An evidence synthesis and economic analysis. Health Technol Assess.

[14] Pluye, P. Robert, E. Cargo, M. Bartlett, G. O’Cathain, A. Griffiths, F. Boardman, F. Gagnon, MP. Rousseau, MC. Proposal: a mixed methods appraisal tool for systematic mixed studies reviews. 2011. Available from: http://mixedmethodsappraisaltoolpublic.pbworks.com/FrontPage. Cited 2018.

[15] Srivastava, A. and S. Thompson, Framework analysis: a qualitative methodology for applied policy research. Journal of Administration and Governance. 2009;4:(2).

[16] Braun V, Clarke V (2006). Using thematic analysis in psychology. Qual Res Psychol.

[17] Atkins L, Francis J, Islam R, O'Connor D, Patey A, Ivers N (2017). A guide to using the theoretical domains framework of behaviour change to investigate implementation problems. Implement Sci.

[18] Michie S, Johnston M, Francis J, Hardeman W, Eccles M (2008). From theory to intervention: mapping theoretically derived behavioural determinants to behaviour change techniques. Appl Psychol.

[19] Cane J, Richardson M, Johnston M, Ladha R, Michie S (2015). From lists of behaviour change techniques (BCTs) to structured hierarchies: comparison of two methods of developing a hierarchy of BCTs. Br J Health Psychol.

[20] Getliffe K, Newton T (2006). Catheter-associated urinary tract infection in primary and community health care. Age & Ageing.

[21] Krein SL, Harrod M, Collier S, Davis KK, Rolle AJ, Fowler KE (2017). A national collaborative approach to reduce catheter-associated urinary tract infections in nursing homes: a qualitative assessment. American Journal of Infection Control.

[22] Krein SL, Kowalski CP, Harrod M, Forman J, Saint S. Barriers to reducing urinary catheter use: a qualitative assessment of a statewide initiative. JAMA Intern Med. 2013;(10):173, 881–6. 10.1001/jamainternmed.2013.105 PMC3665648 23529627

[23] Harrod M, Kowalski CP, Saint S, Forman J, Krein SL (2013). Variations in risk perceptions: a qualitative study of why unnecessary urinary catheter use continues to be problematic. BMC Health Serv Res.

[24] Alexaitis I, Broome B (2014). Implementation of a nurse-driven protocol to prevent catheter-associated urinary tract infections. J Nurs Care Qual.

[25] Andreessen L, Wilde MH, Herendeen P (2012). Preventing catheter-associated urinary tract infections in acute care: the bundle approach. J Nurs Care Qual.

[26] Apisarnthanarak A, Ratz D, Greene MT, Khawcharoenporn T, Weber DJ, Saint S (2017). National survey of practices to prevent health care-associated infections in Thailand: the role of prevention bundles. Am J Infect Control.

[27] Bursle EC, Dyer J, Looke DF, McDougall DA, Paterson DL, Playford EG (2015). Risk factors for urinary catheter associated bloodstream infection. J Infect.

[28] Carter EJ, Pallin DJ, Mandel L, Sinnette C, Schuur JD (2016). Emergency department catheter-associated urinary tract infection prevention: multisite qualitative study of perceived risks and implemented strategies. Infect Control Hospl Epidemiol.

[29] Carter EJ, Pallin DJ, Mandel L, Sinnette C, Schuur JD (2016). A qualitative study of factors facilitating clinical nurse engagement in emergency department catheter-associated urinary tract infection prevention. J Nurs Adm.

[30] Hu FW, Yang DC, Huang CC, Chen CH, Chang CM (2015). Inappropriate use of urinary catheters among hospitalized elderly patients: clinician awareness is key. Geriatr Gerontol Int.

[31] Conner BT, Kelechi TJ, Nemeth LS, Mueller M, Edlund BJ, Krein SL (2013). Exploring factors associated with nurses’ adoption of an evidence-based practice to reduce duration of catheterization. J Nurs Care Qual.

[32] Conway LJ, Pogorzelska M, Larson E, Stone PW (2012). Adoption of policies to prevent catheter-associated urinary tract infections in United States intensive care units. Am J Infect Control.

[33] Crouzet J, Bertrand X, Venier AG, Badoz M, Husson C, Talon D (2007). Control of the duration of urinary catheterization: impact on catheter-associated urinary tract infection. J Hosp Infect.

[34] Dugyon-Escalante J, Yoon J, Lavelle J, Naungayan A (2015). The impact of multidisciplinary team approach to combat catheter associated urinary tract infection in a large government hospital. Am J Infect Control.

[35] Fakih MG, Dueweke C, Meisner S, Berriel-Cass D, Savoy-Moore R, Brach N (2008). Effect of nurse-led multidisciplinary rounds on reducing the unnecessary use of urinary catheterization in hospitalized patients. Infect Control Hosp Epidemiol.

[36] Fakih MG, George C, Edson BS, Goeschel CA, Saint S (2013). Implementing a national program to reduce catheter-associated urinary tract infection: a quality improvement collaboration of state hospital associations, academic medical centers, professional societies, and governmental agencies. Infect Control Hosp Epidemiol.

[37] Gupta SS, Irukulla PK, Shenoy MA, Nyemba V, Yacoub D, Kupfer Y. Successful strategy to decrease indwelling catheter utilization rates in an academic medical intensive care unit, Am J Infect Control. 2017;45(12):1349–55. 10.1016/j.ajic.2017.06.020 28844376

[38] Mann P, Vandygriff C, Kingsberry L, Horne R, Strelczyk K. Catheter-associated urinary tract infection (CAUTI): a significant case for concern. Am J Infect Control. 2013;1:–S124.

[39] Murphy C, Prieto J, Fader M (2015). “It’s easier to stick a tube in”: a qualitative study to understand clinicians’ individual decisions to place urinary catheters in acute medical care. BMJ Quality & Safety.

[40] Patrizzi K, Fasnacht A, Manno M (2009). A collaborative, nurse-driven initiative to reduce hospital-acquired urinary tract infections. J Emerg Nurs.

[41] Smith LC, Peyton J, Krout K, Cox CA, Huber KL (2015). Decreasing the rate of catheter-associated urinary tract infections through a nurse-driven intervention. J Burn Care Res.

[42] Fakih MG, Pena ME, Shemes S, Rey J, Berriel-Cass D, Szpunar SM (2010). Effect of establishing guidelines on appropriate urinary catheter placement. Acad Emerg Med.

[43] Trautner BW, Petersen NJ, Hysong SJ, Horwitz D, Kelly PA, Naik AD (2014). Am J Infect Control.

[44] Kolonoski P, Stanley K, Anderson K (2012). An interdisciplinary approach toward reducing the incidence of catheter-associated urinary tract infections in a post-acute facility. Am J Infect Control.

[45] Grol R, Grimshaw J (2003). From best evidence to best practice: effective implementation of change in patients’ care. Lancet.

