# Supplementary material for: Reducing catheter-associated urinary tract infections: a systematic review of barriers and facilitators and strategic behavioural analysis of interventions
Source: Implement Sci. 2020 Jul 6;15:44. doi: 10.1186/s13012-020-01001-2 (PMC7336619; doi:10.1186/s13012-020-01001-2)
Supplement: Supplementary file 1 — Additional file 1. Labels, definitions and examples of COM-B and Theoretical Domains Framework [file 13012_2020_1001_MOESM1_ESM.docx]

**Additional file 1. Labels, definitions and examples of COM-B and Theoretical Domains Framework**

**COM-B model**

| **COM-B model component**  Definition | ***Example*** |
| --- | --- |
| **Physical capability**  Physical skill, strength or stamina | *Having the skill to take a blood sample* |
| **Psychological capability**  Knowledge or psychological skills, strength or stamina to engage in the necessary mental processes | *Understanding the impact of CO^2^ on the environment* |
| **Physical opportunity**  Opportunity afforded by the environment involving time, resources, locations, cues, physical ‘affordance’ | *Being able to go running because one owns appropriate shoes* |
| **Social opportunity**  Opportunity afforded by interpersonal influences, social cues and cultural norms that influence the way that we think about things, e.g. the words and concepts that make up our language | *Being able to smoke in the house of someone who smokes but not in the middle of a boardroom meeting* |
| **Reflective motivation**  Reflective processes involving plans (self-conscious intentions) and evaluations (beliefs about what is good and bad) | *Intending to stop smoking* |
| **Automatic motivation**  Automatic processes involving emotional reactions, desires (wants and needs), impulses, inhibitions, drive states and reflex responses | *Feeling anticipated pleasure at the prospect of eating a piece of chocolate cake* |

##

**Theoretical domains framework**

| **Domain**  Definition | **Theoretical constructs represented within each domain** | ***Interview questions**** |
| --- | --- | --- |
| **Knowledge**  An awareness of the existence of something | Knowledge (including knowledge of condition /scientific rationale); procedural knowledge; knowledge of task environment | *Do you know about x?* |
| **Skills**  An ability or proficiency acquired through practice | Skills; skills development; competence; ability; interpersonal skills; practice; skill assessment | *Do you know how to do x?* |
| **Memory, attention and decision processes**  The ability to retain information, focus selectively on aspects of the environment and choose between two or more alternatives | Memory; attention; attention control; decision making; cognitive overload / tiredness | *Is x something you usually do?* |
| **Behavioural regulation**  Anything aimed at managing or changing objectively observed or measured actions | Self-monitoring; breaking habit; action planning | *Do you have systems that you could use for monitoring whether or not you have carried x?* |
| **Social/professional role and identity**  A coherent set of behaviours and displayed personal qualities of an individual in a social or work setting | Professional identity; professional role; social identity; identity; professional boundaries; professional confidence; group identity; leadership; organisational commitment | *Is doing x compatible or in conflict with professional standards/identity?* |
| **Beliefs about capabilities**  Acceptance of the truth, reality, or validity about an ability, talent, or facility that a person can put to constructive use | Self-confidence; perceived competence; self-efficacy; perceived behavioural control; beliefs; self-esteem; empowerment; professional confidence | *How difficult or easy is it for you to do x?* |
| **Optimism**  The confidence that things will happen for the best or that desired goals will be attained | Optimism; pessimism; unrealistic optimism; identity | *How confident are you that the problem of implementing x will be solved?* |
| **Beliefs about consequences**  Acceptance of the truth, reality, or validity about outcomes of a behaviour in a given situation) | Beliefs; outcome expectancies; characteristics of outcome expectancies; anticipated regret; consequents | *What do you think will happen if you do x?* |
| **Intentions**  A conscious decision to perform a behaviour or a resolve to act in a certain way | Stability of intentions; stages of change model; transtheoretical model and stages of change | *Have they made a decision to do x?* |
| **Goals**  Mental representations of outcomes or end states that an individual wants to achieve | Goals (distal / proximal) ; goal priority; goal / target setting; goals (autonomous / controlled); action planning; implementation intention | *How much do they want to do x?* |
| **Reinforcement**  Increasing the probability of a response by arranging a dependent relationship, or contingency, between the response and a given stimulus | Rewards (proximal / distal, valued / not valued, probable / improbable); incentives; punishment; consequents; reinforcement; contingencies; sanctions | *Are there incentives to do x?* |
| **Emotion**  A complex reaction pattern, involving experiential, behavioural, and physiological elements, by which the individual attempts to deal with a personally significant matter or event | Fear; anxiety; affect; stress; depression; positive / negative affect; burn-out | *Does doing x evoke an emotional response?* |
| **Environmental context and resources**  Any circumstance of a person's situation or environment that discourages or encourages the development of skills and abilities, independence, social competence, and adaptive behaviour | Environmental stressors ; resources / material resources ; organisational culture /climate ; salient events / critical incidents; person x environment interaction; barriers and facilitators | *To what extent do physical or resource factors facilitate or hinder x?* |
| **Social influences**  Those interpersonal processes that can cause individuals to change their thoughts, feelings, or behaviours | Social pressure; social norms; group conformity; social comparisons; group norms; social support; power; intergroup conflict; alienation; group identity; modelling | *To what extent do social influences facilitate or hinder x?* |
| * Summarised from Michie et al. (2005) | | |
