# Supplementary material for: Reducing catheter-associated urinary tract infections: a systematic review of barriers and facilitators and strategic behavioural analysis of interventions
Source: Implement Sci. 2020 Jul 6;15:44. doi: 10.1186/s13012-020-01001-2 (PMC7336619; doi:10.1186/s13012-020-01001-2)
Supplement: Supplementary file 2 — Additional file 2. Behaviour Change Wheel labels, definitions and examples [file 13012_2020_1001_MOESM2_ESM.docx]

# Additional file 2. Behaviour Change Wheel labels, definitions and examples

**Intervention functions**

| **Intervention function** | **Definition** | ***Example of intervention function*** |
| --- | --- | --- |
| **Education** | Increasing knowledge or understanding | *Providing information to promote healthy eating* |
| **Persuasion** | Using communication to induce positive or negative feelings or stimulate action | *Using imagery to motivate increases in physical activity* |
| **Incentivisation** | Creating an expectation of reward | *Using prize draws to induce attempts to stop smoking* |
| **Coercion** | Creating an expectation of punishment or cost | *Raising the financial cost to reduce excessive alcohol consumption* |
| **Training** | Imparting skills | *Advanced driver training to increase safe driving* |
| **Restriction** | Using rules to reduce the opportunity to engage in the target behaviour (or to increase the target behaviour by reducing the opportunity to engage in competing behaviours) | *Prohibiting sales of solvents to people under 18 to reduce use for intoxication* |
| **Environmental restructuring** | Changing the physical or social context | *Providing on-screen prompts for GPs to ask about smoking behaviour* |
| **Modelling** | Providing an example for people to aspire to or imitate | *Using TV drama scenes involving safe-sex practices to increase condom use* |
| **Enablement** | Increasing means/reducing barriers to increase capability (beyond education and training) or opportunity (beyond environmental restructuring) | *Behavioural support for smoking cessation, medication for cognitive deficits, surgery to reduce obesity, prostheses to promote physical activity* |

**Policy category definitions**

| **Policy Category** | **Definition** | **Example** |
| --- | --- | --- |
| **Communication/ marketing** | Using print, electronic, telephonic or broadcast media | *Conducting mass media campaigns* |
| **Guidelines** | Creating documents that recommend or mandate practice. This includes all changes to service provision | *Producing and disseminating treatment protocols* |
| **Fiscal measures** | Using the tax system to reduce or increase the financial cost | *Increasing duty or increasing anti-smuggling activities* |
| **Regulation** | Establishing rules or principles of behaviour or practice | *Establishing voluntary agreements on advertising* |
| **Legislation** | Making or changing laws | *Prohibiting sale or use* |
| **Environmental/social planning** | Designing and/or controlling the physical or social environment | *Using town planning* |
| **Service provision** | Delivering a service | *Establishing support services in workplaces, communities etc.* |
