# Supplementary material for: Reducing catheter-associated urinary tract infections: a systematic review of barriers and facilitators and strategic behavioural analysis of interventions
Source: Implement Sci. 2020 Jul 6;15:44. doi: 10.1186/s13012-020-01001-2 (PMC7336619; doi:10.1186/s13012-020-01001-2)
Supplement: Supplementary file 3 — Additional file 3. BCW matrices [file 13012_2020_1001_MOESM3_ESM.docx]

**Additional file 3. BCW matrices**

**Links between COM-B and intervention functions**

**Links between intervention functions and policy categories**
