# Supplementary material for: Reducing catheter-associated urinary tract infections: a systematic review of barriers and facilitators and strategic behavioural analysis of interventions
Source: Implement Sci. 2020 Jul 6;15:44. doi: 10.1186/s13012-020-01001-2 (PMC7336619; doi:10.1186/s13012-020-01001-2)
Supplement: Supplementary file 5 — Additional file 5. Electronic search strategies [file 13012_2020_1001_MOESM5_ESM.docx]

**Additional file 5. Electronic search strategies**

**Systematic review of barriers and facilitators of catheter associated urinary tract infections (CAUTI) behaviours in secondary care and nursing homes**

| 1. (urinary adj1 tract adj1 infection*).mp. [mp=ti, ab, hw, tn, ot, dm, mf, dv, kw, fx, nm, kf, px, rx, ui, sy, tc, id, tm] |  |
| --- | --- |
| 2. UTI.mp. [mp=ti, ab, hw, tn, ot, dm, mf, dv, kw, fx, nm, kf, px, rx, ui, sy, tc, id, tm] |  |
| 3. CAUTI.mp. [mp=ti, ab, hw, tn, ot, dm, mf, dv, kw, fx, nm, kf, px, rx, ui, sy, tc, id, tm] |  |
| 4. (acute adj1 kidney adj1 injury).mp. [mp=ti, ab, hw, tn, ot, dm, mf, dv, kw, fx, nm, kf, px, rx, ui, sy, tc, id, tm] |  |
| 5. (health$ adj1 associated adj1 infection).mp. [mp=ti, ab, hw, tn, ot, dm, mf, dv, kw, fx, nm, kf, px, rx, an, ui, sy, tc, id, tm] |  |
| 6. (secondary adj1 care).mp. [mp=ti, ab, hw, tn, ot, dm, mf, dv, kw, fx, nm, kf, px, rx, ui, sy, tc, id, tm] |  |
| 7. hospi$.mp. [mp=ti, ab, hw, tn, ot, dm, mf, dv, kw, fx, nm, kf, px, rx, ui, sy, tc, id, tm] |  |
| 8. (nursing adj1 home).mp. [mp=ti, ab, hw, tn, ot, dm, mf, dv, kw, fx, nm, kf, px, rx, ui, sy, tc, id, tm] |  |
| 9. (residential adj1 care).mp. [mp=ti, ab, hw, tn, ot, dm, mf, dv, kw, fx, nm, kf, px, rx, ui, sy, tc, id, tm] |  |
| 10. catheter*.mp. [mp=ti, ab, hw, tn, ot, dm, mf, dv, kw, fx, nm, kf, px, rx, ui, sy, tc, id, tm] |  |
| 11. 1 or 2 or 3 or 4 or 5 |  |
| 12. 6 or 7 or 8 or 9 |  |
| 13. 10 and 11 and 12 |  |
| 14. barrier$.mp. [mp=ti, ab, hw, tn, ot, dm, mf, dv, kw, fx, nm, kf, px, rx, ui, sy, tc, id, tm] |  |
| 15. facilitat$.mp. [mp=ti, ab, hw, tn, ot, dm, mf, dv, kw, fx, nm, kf, px, rx, ui, sy, tc, id, tm] |  |
| 16. enable$.mp. [mp=ti, ab, hw, tn, ot, dm, mf, dv, kw, fx, nm, kf, px, rx, ui, sy, tc, id, tm] |  |
| 17. lever$.mp. [mp=ti, ab, hw, tn, ot, dm, mf, dv, kw, fx, nm, kf, px, rx, ui, sy, tc, id, tm] |  |
| 18. obstacle$.mp. [mp=ti, ab, hw, tn, ot, dm, mf, dv, kw, fx, nm, kf, px, rx, ui, sy, tc, id, tm] |  |
| 19. influen$.mp. [mp=ti, ab, hw, tn, ot, dm, mf, dv, kw, fx, nm, kf, px, rx, ui, sy, tc, id, tm] |  |
| 20. drive*.mp. [mp=ti, ab, hw, tn, ot, dm, mf, dv, kw, fx, nm, kf, px, rx, ui, sy, tc, id, tm] |  |
| 21. determin$.mp. [mp=ti, ab, hw, tn, ot, dm, mf, dv, kw, fx, nm, kf, px, rx, ui, sy, tc, id, tm] |  |
| 22. factor$.mp. [mp=ti, ab, hw, tn, ot, dm, mf, dv, kw, fx, nm, kf, px, rx, ui, sy, tc, id, tm] |  |
| 23. 14 or 15 or 16 or 17 or 18 or 19 or 20 or 21 or 22 |  |
| 24. 13 and 23 |  |
| 25. limit 24 to english language |  |
| 26. limit 25 to human |  |
| 27. limit 26 to yr="1995 -Current" |  |
| 28. limit 27 to humans |  |

**Systematic review of barriers and facilitators of catheter associated urinary tract infections (CAUTI) behaviours in primary and community care**

| 1. (urinary adj1 tract adj1 infection*).mp. [mp=ti, ab, hw, tn, ot, dm, mf, dv, kw, fx, nm, kf, px, rx, ui, sy, tc, id, tm] | | |  |
| --- | --- | --- | --- |
| 2. UTI.mp. [mp=ti, ab, hw, tn, ot, dm, mf, dv, kw, fx, nm, kf, px, rx, ui, sy, tc, id, tm] | | |  |
| 3. CAUTI.mp. [mp=ti, ab, hw, tn, ot, dm, mf, dv, kw, fx, nm, kf, px, rx, ui, sy, tc, id, tm] | | |  |
| 4. (acute adj1 kidney adj1 injury).mp. [mp=ti, ab, hw, tn, ot, dm, mf, dv, kw, fx, nm, kf, px, rx, ui, sy, tc, id, tm] | | |  |
| 5. (health$ adj1 associated adj1 infection).mp. [mp=ti, ab, hw, tn, ot, dm, mf, dv, kw, fx, nm, kf, px, rx, ui, sy, tc, id, tm] | | |  |
| 6. (primary adj1 care).mp. [mp=ti, ab, hw, tn, ot, dm, mf, dv, kw, fx, nm, kf, px, rx, ui, sy, tc, id, tm] | | |  |
| 7. (general adj1 practi$).mp. [mp=ti, ab, hw, tn, ot, dm, mf, dv, kw, fx, nm, kf, px, rx, ui, sy, tc, id, tm] | | |  |
| 8. (primary adj1 health adj1 care).mp. [mp=ti, ab, hw, tn, ot, dm, mf, dv, kw, fx, nm, kf, px, rx, ui, sy, tc, id, tm] | | |  |
| 9. (primary adj1 healthcare).mp. [mp=ti, ab, hw, tn, ot, dm, mf, dv, kw, fx, nm, kf, px, rx, ui, sy, tc, id, tm] | | |  |
| 10. GP.mp. [mp=ti, ab, hw, tn, ot, dm, mf, dv, kw, fx, nm, kf, px, rx, ui, sy, tc, id, tm] | | |  |
| 11. (family adj1 physician$).mp. [mp=ti, ab, hw, tn, ot, dm, mf, dv, kw, fx, nm, kf, px, rx, ui, sy, tc, id, tm] | | |  |
| 12. (primary adj1 care adj1 physician$).mp. [mp=ti, ab, hw, tn, ot, dm, mf, dv, kw, fx, nm, kf, px, rx, ui, sy, tc, id, tm] | | |  |
| 13. communit$.mp. [mp=ti, ab, hw, tn, ot, dm, mf, dv, kw, fx, nm, kf, px, rx, ui, sy, tc, id, tm] | | |  |
| 14. continence nurse$.mp. [mp=ti, ab, hw, tn, ot, dm, mf, dv, kw, fx, nm, kf, px, rx, ui, sy, tc, id, tm] | | |  |
| 15. (home adj1 care).mp. [mp=ti, ab, hw, tn, ot, dm, mf, dv, kw, fx, nm, kf, px, rx, an, ui, sy, tc, id, tm] | | |  |
| 16. (home adj1 nurs$).mp. [mp=ti, ab, hw, tn, ot, dm, mf, dv, kw, fx, nm, kf, px, rx, an, ui, sy, tc, id, tm] | | |  |
| 17. catheter*.mp. [mp=ti, ab, hw, tn, ot, dm, mf, dv, kw, fx, nm, kf, px, rx, ui, sy, tc, id, tm] | | |  |
| 18. 1 or 2 or 3 or 4 or 5 | | |  |
| 19. 6 or 7 or 8 or 9 or 10 or 11 or 12 or 13 or 14 or 15 or 16 or 17 | | |  |
| 20. 17 and 18 and 19 | | |  |
| 21. barrier$.mp. [mp=ti, ab, hw, tn, ot, dm, mf, dv, kw, fx, nm, kf, px, rx, ui, sy, tc, id, tm] | | |  |
| 22. facilitat$.mp. [mp=ti, ab, hw, tn, ot, dm, mf, dv, kw, fx, nm, kf, px, rx, ui, sy, tc, id, tm] | | |  |
| 23. enable$.mp. [mp=ti, ab, hw, tn, ot, dm, mf, dv, kw, fx, nm, kf, px, rx, ui, sy, tc, id, tm] | | |  |
| 24. lever$.mp. [mp=ti, ab, hw, tn, ot, dm, mf, dv, kw, fx, nm, kf, px, rx, ui, sy, tc, id, tm] | | |  |
| 25. obstacle$.mp. [mp=ti, ab, hw, tn, ot, dm, mf, dv, kw, fx, nm, kf, px, rx, ui, sy, tc, id, tm] | | |  |
| 26. influen$.mp. [mp=ti, ab, hw, tn, ot, dm, mf, dv, kw, fx, nm, kf, px, rx, ui, sy, tc, id, tm] | | |  |
| 27. drive*.mp. [mp=ti, ab, hw, tn, ot, dm, mf, dv, kw, fx, nm, kf, px, rx, ui, sy, tc, id, tm] | | |  |
| 28. determin$.mp. [mp=ti, ab, hw, tn, ot, dm, mf, dv, kw, fx, nm, kf, px, rx, ui, sy, tc, id, tm] | | |  |
| 29. factor$.mp. [mp=ti, ab, hw, tn, ot, dm, mf, dv, kw, fx, nm, kf, px, rx, ui, sy, tc, id, tm] | | |  |
| 30. 21 or 22 or 23 or 24 or 25 or 26 or 27 or 28 or 29 | | |  |
| 31. 20 and 30 | | |  |
| 32. limit 31 to english language | | |  |
| 33. limit 32 to human | | |  |
| 34. limit 33 to yr="1995 -Current" | | |  |
| 35. limit 34 to humans |  |  |  |
