# Supplementary material for: Reducing catheter-associated urinary tract infections: a systematic review of barriers and facilitators and strategic behavioural analysis of interventions
Source: Implement Sci. 2020 Jul 6;15:44. doi: 10.1186/s13012-020-01001-2 (PMC7336619; doi:10.1186/s13012-020-01001-2)
Supplement: Supplementary file 6 — Additional file 6. Merged Theoretical Domain Framework x Behaviour Change Techniques matrices [file 13012_2020_1001_MOESM6_ESM.docx]

# Additional file 6. Merged Theoretical Domain Framework x Behaviour Change Techniques matrices

| **COM-B component** | **TDF Domain** | **BCTs-**  **CANE et al 2015** | **BCTs-**  **MICHIE et al 2008** | **BCTs-**  **MERGED** |
| --- | --- | --- | --- | --- |
| **CAPABILITY**  **(Psychological)** | **Knowledge** | Information on health consequences | Information on health consequences | Information on health consequences |
|  |  | Biofeedback | + Information on social/ environmental consequences | Biofeedback |
|  |  | Antecedents | + Information emotional consequences | Antecedents |
|  |  | Feedback on behaviour | + Salience of consequences | Feedback on behaviour |
|  |  |  |  | Information on social/ environmental consequences |
|  |  |  |  | Information emotional consequences |
|  |  |  |  | Salience of consequences |
|  | **Memory, Attention, Decision Making** | NONE | + Self-monitoring of behaviour | Self-monitoring of behaviour |
|  |  |  | + Self-monitoring of outcome of behaviour | Self-monitoring of outcome of behaviour |
|  |  |  | + Action planning | Action planning |
|  |  |  | + Prompts and cues | Prompts and cues |
|  | **Behavioural Regulation** | Self-monitoring of behaviour | +Goal setting (behaviour) | Self-monitoring of behaviour |
|  |  |  | + Goal setting (outcome) | Goal setting (outcome)  Goal setting behaviour |
|  |  |  | + Behavioural contract | Behavioural contract |
|  |  |  | + Action planning (including implementation intentions) | Action planning (including  implementation intentions) |
|  |  |  | + prompts/cues | Prompts/cues |
| **CAPABILITY (Physical/ Psychological)** | **Skills** | Graded tasks | Graded tasks | Graded tasks |
|  |  | Behavioural rehearsal/practice | Behavioural rehearsal/practice | Behavioural rehearsal/practice |
|  |  | Habit reversal | +goal setting (outcome) | Habit reversal |
|  |  | Body changes | + goal setting (behaviour) | Body changes |
|  |  | Habit formation | +monitoring by others without feedback | Habit formation |
|  |  |  | +self-monitoring | goal setting (outcome) |
|  |  |  | +reward (outcome) | goal setting (behaviour) |
|  |  |  | + self-reward | monitoring by others without feedback |
|  |  |  | +Incentive | self-monitoring |
|  |  |  | +Material reward | reward (outcome) |
|  |  |  | +Non-specific reward | self-reward |
|  |  |  | +demonstration of the behaviour (modelling) | Incentive |
|  |  |  | +generalisation of target behaviour | Material reward |
|  |  |  |  | Non-specific reward |
|  |  |  |  | demonstration of the behaviour (modelling) |
|  |  |  |  | generalisation of target behaviour |
| **OPPORTUNITY**  **(social)** | **Social Influences** | Social comparison | Social processes of encouragement, pressure or support | Social comparison |
|  |  | Social support (unspecified) | +demonstration of the behaviour (modelling) | Social support (unspecified) |
|  |  | Social support (emotional) |  | Social support (emotional) |
|  |  | Social support (practical) |  | Social support (practical) |
|  |  | Information about others’ approval |  | Information about others’ approval |
|  |  | Vicarious consequences/ reinforcement |  | Vicarious consequences/ reinforcement |
|  |  | Restructuring the social environment |  | Restructuring the social environment |
|  |  | Identification of self as a role model |  | Identification of self as a role model |
|  |  | Social Reward |  | Social Reward |
|  |  |  |  | Demonstration of the behaviour |
| **OPPORTUNITY (physical)** | **Environmental Context and Resources** | Restructuring the physical environment | +Environmental changes (e.g. objects to facilitate behaviour) i.e. adding objects to the environment | Restructuring the physical environment |
|  |  | Discriminative (learned) cue |  | Discriminative (learned) cue |
|  |  | Prompts/ Cues |  | Prompts/ Cues |
|  |  | Avoidance/ changing exposure to cues for the behaviour  Restructuring the social environment |  | Avoidance/ changing exposure to cues for the behaviour  Restructuring the social environment |
|  |  |  |  | Adding objects to the environment |
| **MOTIVATION (Reflective)** | **Beliefs about consequences** | Information about emotional consequences | +Self-monitoring of behaviour | Information about emotional consequences |
|  |  | Salience of consequences | +Self-monitoring of outcome of behaviour | Salience of consequences |
|  |  | Covert Sensitization | +Information on health consequences | Covert Sensitization |
|  |  | Anticipated regret | +Feedback on behaviour | Anticipated regret |
|  |  | Information about social/ environmental consequences | +Biofeedback | Information about social/ environmental consequences |
|  |  | Pros and Cons | +Feedback on outcome(s) of behaviour | Pros and Cons |
|  |  | Vicarious reinforcement | +Persuasive communication (Credible source) | Vicarious reinforcement |
|  |  | Threat | Information on social/ environmental consequences | Threat |
|  |  | Comparative imagining of future outcomes | Salience of consequences | Comparative imagining of future outcomes |
|  |  |  | Information emotional consequences | Self-monitoring of behaviour |
|  |  |  |  | Self-monitoring of outcome of behaviour |
|  |  |  |  | Information on health consequences |
|  |  |  |  | Feedback on behaviour |
|  |  |  |  | Biofeedback |
|  |  |  |  | Feedback on outcome(s) of behaviour |
|  |  |  |  | Persuasive communication (Credible source) |
|  | **Beliefs about capabilities** | Verbal persuasion to boost self-efficacy | Motivational interviewing (i.e. verbal persuasion to boost self-efficacy) | Verbal persuasion to boost self-efficacy |
|  |  | Focus on past success | +Self-monitoring of behaviour | Focus on past success |
|  |  |  | +Self-monitoring of outcome of behaviour | Self-monitoring of behaviour |
|  |  |  | + Graded tasks | Self-monitoring of outcome of behaviour |
|  |  |  | + Problem solving | Graded tasks |
|  |  |  | + Goal setting (Behaviour) | Problem solving |
|  |  |  | + Goal setting (outcome) | Goal setting (Behaviour) |
|  |  |  | +coping skills | Goal setting (outcome) |
|  |  |  | + behavioural practice/rehearsal | Coping skills |
|  |  |  | +Social support (unspecified) | Behavioural practice/rehearsal |
|  |  |  | +Social support (emotional) | Social support (unspecified) |
|  |  |  | +Social support (practical) | Social support (emotional) |
|  |  |  | + feedback (behaviour) | Social support (practical) |
|  |  |  | + feedback (outcome) | Feedback (behaviour) |
|  |  |  | +Self-talk | feedback (outcome) |
|  |  |  |  | Self-talk |
|  | **Optimism** | Verbal persuasion to boost self-efficacy | NONE | Verbal persuasion to boost self-efficacy |
|  | **Social professional role/ identity** | NONE | +Social support (unspecified) | Social support (unspecified) |
|  |  |  | +Social support (emotional) | Social support (emotional) |
|  |  |  | +Social support (practical) | Social support (practical) |
|  | **Intentions** | Commitment | Commitment | Commitment |
|  |  | Behavioural contract | Behavioural contract | Behavioural contract |
|  | **Goals** | Goal setting (outcome) | Goal setting (outcome) | Goal setting (outcome) |
|  |  | Goal setting (behaviour) | Goal setting (behaviour) | Goal setting (behaviour) |
|  |  | Review of outcome goal(s) | + Problem solving | Review of outcome goal(s) |
|  |  | Review behaviour goals | +Social support (unspecified) | Review behaviour goals |
|  |  | Action planning (Implementation Intentions) | +Social support (emotional) | Action planning (Implementation Intentions) |
|  |  |  | +Social support (practical) | Problem solving |
|  |  |  | + feedback (behaviour) | Social support (unspecified) |
|  |  |  | + feedback (outcome) | Social support (emotional) |
|  |  |  | +Motivational interviewing (i.e. verbal persuasion to boost self-efficacy) | Social support (practical) |
|  |  |  | +Stress management (i.e. reduce negative emotions; conserving mental resources) | Feedback (behaviour) |
|  |  |  | + persuasive communication (i.e. verbal persuasion to boost self-efficacy) | feedback (outcome) |
|  |  |  | +reward (outcome) | Verbal persuasion to boost self-efficacy |
|  |  |  | + self-reward | Reduce negative emotions |
|  |  |  | +Incentive | Conserving mental resources |
|  |  |  | +Material reward | Reward (outcome) |
|  |  |  | +Non-specific reward | Self-reward |
|  |  |  |  | Non-specific reward |
| **MOTIVATION**  **(Automatic)** | **Reinforcement** | Threat | NONE | Threat |
|  |  | Self-reward |  | Self-reward |
|  |  | Differential reinforcement |  | Differential reinforcement |
|  |  | Incentive |  | Incentive |
|  |  | Thinning |  | Thinning |
|  |  | Negative reinforcement |  | Negative reinforcement |
|  |  | Shaping |  | Shaping |
|  |  | Counter conditioning |  | Counter conditioning |
|  |  | Discrimination training |  | Discrimination training |
|  |  | Material reward |  | Material reward |
|  |  | Non-specific reward |  | Non-specific reward |
|  |  | Response cost |  | Response cost |
|  |  | Anticipation of future rewards or removal of punishment |  | Anticipation of future rewards or removal of punishment |
|  |  | Punishment |  | Punishment |
|  |  | Extinction |  | Extinction |
|  |  | Classical Conditioning |  | Classical Conditioning |
|  | **Emotions** | Reduce negative emotions | Reduce negative emotions | Reduce negative emotions |
|  |  | Information about emotional consequences | +Conserving mental resources | Information about emotional consequences |
|  |  | Self-assessment of affective consequences |  | Self-assessment of affective consequences |
|  |  | Social support (emotional) |  | Social support (emotional) |
|  |  |  |  | Conserving mental resources |
