# Supplementary material for: Reducing catheter-associated urinary tract infections: a systematic review of barriers and facilitators and strategic behavioural analysis of interventions
Source: Implement Sci. 2020 Jul 6;15:44. doi: 10.1186/s13012-020-01001-2 (PMC7336619; doi:10.1186/s13012-020-01001-2)
Supplement: Supplementary file 7 — Additional file 7. Flow of information through the systematic review [file 13012_2020_1001_MOESM7_ESM.docx]

# Additional file 7. Flow of information through the systematic review

**Secondary care and nursing homes**

**Primary and community care**
