# Supplementary material for: Reducing catheter-associated urinary tract infections: a systematic review of barriers and facilitators and strategic behavioural analysis of interventions
Source: Implement Sci. 2020 Jul 6;15:44. doi: 10.1186/s13012-020-01001-2 (PMC7336619; doi:10.1186/s13012-020-01001-2)
Supplement: Supplementary file 9 — Additional file 9. Summary of barriers to and facilitators of CAUTI-related behaviours [file 13012_2020_1001_MOESM9_ESM.docx]

**Additional file 9. Summary of barriers to and facilitators of CAUTI-related behaviours**

| **COM-B** | **Theme** | **Frequency**  **(n= 25 studies max)** | **Barrier/ facilitator/ Mixed** | **Behavioural phase** | **Example quote(s)**  **[Setting; Study ID]** |
| --- | --- | --- | --- | --- | --- |
| CAPABILITY (PSYCHOLOGICAL) | TDF Domain: Knowledge | | | | |
|  | Lack of knowledge of clinical guidelines and local procedural documents | 3 | Barrier | All | ‘Nurses lack of knowledge of evidence-based guidelines for: routine catheter maintenance; urinary catheter indications; bladder scanning; and intermittent catheterization.’ [Secondary Care; S2]  ‘40% of the Burn ICU nursing staff was not aware of the nurse driven catheter removal protocol that exists within our hospital.’ [Secondary Care; S19] |
|  | Lack of information regarding placement and duration of catheter insertion | 2 | Barrier | Insertion, post-insertion maintenance | ‘Physicians are often unaware that urinary catheterization  has been excessively prolonged in their patients’ [Secondary Care; S11]  ‘physicians were often not aware that patients had an indwelling catheter’ [Tertiary Care; T1] |
|  | Lack of awareness of the risks associated with use of urinary catheters | 2 | Barrier | All | “… so, it’s just making them [nurses] understand that  there is a relationship between bladder infections and urinary tract infections and [urinary] catheter days…” [Secondary Care; S1] |
|  | Lack of knowledge of how to manage patients without catheterisation | 1 | Barrier | Pre-insertion | ‘Not knowing how to manage critically ill patients in ICU without using indwelling urinary catheter.’ [Secondary Care; S15] |
|  | Knowledge of how to manage bacterial infections resulting from urinary catheterisation | 1 | Facilitator | Post-insertion maintenance | ‘Knowledge of how to manage catheter-associated bacteriuria: “The mean knowledge score was 57.5%, or slightly more than one-half of the questions answered correctly. The mean knowledge score was 57.5%, or slightly more than one-half of the questions answered correctly.”’ [Tertiary Care; T3] |
|  | TDF Domain: Memory, attention, decision processes | | | | |
|  | Pre-emptively deciding to insert a catheter due to likely subsequent catheterisation | 3 | Barrier | Insertion | ‘Also, I think at the back of my mind is the likelihood is that they’re going to get catheterised in the near future any way when they hit the wards for a management reason’ [Secondary Care; S17] |
|  | Catheterisation decisions based on non-medical criteria | 1 | Barrier | Pre-insertion | ‘Nonmedical criteria often determined urinary catheter placement decisions, with catheters being used to manage patients with incontinence’ [Secondary Care; S6] |
|  | Patient symptoms prompt investigation and treatment of possible CAUTI | 1 | Facilitator | Post-insertion maintenance | ‘I usually order a urine culture on catheterized patients when there is a change in urine colour, cloudiness, or odour.’ [Tertiary care; T3] |
|  | Absence of standardised CAUTI diagnostic criteria to help decision-making | 1 | Barrier | Post-insertion maintenance | lack of clear, standardised criteria used to define CAUTI, to distinguish between symptomatic and asymptomatic CAUTI and to report outcomes’ [Primary Care; p1] |
|  | Reminders and prompts | 1 | Facilitator | Removal | ‘Hospital epidemiologist in post was significantly and positively associated with: i) Urinary catheter reminder or stop-order/nurse- initiated catheter discontinuation’ [Secondary Care; S4] |
| CAPABILITY (PSYCHOLOGICAL) | TDF Domain: Behavioural regulation | | | | |
|  | Audit and feedback on CAUTI metrics | 2 | Facilitator | All | ‘One hospital even made it a point to collect urinary tract infection (UTI) data on patients seven days post discharge to use as evidence for their staff that urinary catheters (and hence their actions related to the use of urinary catheters) do cause infections’ [Secondary Care; S1] |
|  | Inconsistent monitoring of compliance with guidelines | 1 | Barrier | All | ‘Inconsistent monitoring of compliance with evidence-based guidelines.’ [Secondary Care; S2] |
| CAPABILITY (PHYSICAL) | TDF Domain: Skills | | | | |
|  | Poor urinary catheter insertion technique | 2 | Barrier | Insertion | ‘Urinary catheters were being inserted by nursing staff that did not demonstrate proper insertion technique and by medical students and residents who were untrained in catheter placement’ [Secondary Care; S6] |
| OPPORTUNITY (PHYSICAL) | TDF Domain: Environmental context and resources | | | | |
|  | Limited and inconsistent documentation and records | 6 | Barrier | All | ‘Lack of medical documentation for use of urinary catheters was significantly associated with inappropriate catheter use’ [Secondary Care; S8]  The level of detail recorded was variable both within and between service groups. Of the patients with a CAUTI, the date diagnosed, method of diagnosis (e.g. urine culture) and antibiotic treatment given were recorded in over 85% of cases, although the causative organism was reported in <40%. However, it was rarely possible to confirm whether the CAUTI was symptomatic or asymptomatic. [Primary Care; P1] |
|  | Transitions of care | 5 | Barrier | All | ‘Transferences to ward from intensive care unit accounted for 6% of inappropriate catheterisations’ [Secondary Care; S13]  ‘Staff turnover barrier to implementation of CAUTI reduction program’ [Care homes; C1] |
|  | Lack of time to perform alternatives to urinary catheterisation | 3 | Barrier | Pre-insertion | ‘Being too busy to be able to assist a patient to the bathroom’ [Secondary Care; S1] |
|  | Unavailability of medical alternatives to urinary catheterisation | 2 | Barrier | Pre-insertion | ‘Lack of medical alternatives, (e.g., a bladder scanner that could help determine the need for a catheter) that may be contributing to these decisions’ [Secondary Care; S1] |
|  | Choice and availability of urinary catheters | 2 | Mixed | Pre-insertion | ‘Supply of urinary catheters being available in bedside supply carts added convenience and may have influenced their decision to insert one’ [Secondary Care; S18]  ‘In acute care catheter availability was often limited to samples supplied by company representatives or the limited supply held in hospital pharmacies. In the community availability could be limited by a formulary. Of the prescribing nurses, 54% had unlimited choice of catheters, while 43% were restricted in their choice.’ [Secondary care; Community Care; SC20] |
| OPPORTUNITY (SOCIAL) | TDF Domain: Social influences | | | | |
|  | Requests from patients and their carers | 5 | Barrier | Pre-insertion | ‘Pressure of requests by the patient or the family for catheters: “The family says, ‘Well my mom really needs it in. . . . Mom can’t get up, mom can’t walk, she’s incontinent [of urine]’ [Secondary Care; S1] |
|  | Lack of peer support and buy-in | 4 | Barrier | All | ‘Facility leads new to their positions often found it hard to gain buy-in, mainly due to a lack of relationship with the staff’ [Care Homes; C1]  ‘As a charge nurse explained: “If you don’t have the doctors on board, you’re just going to be beating your head against the wall.’ [Secondary Care; S1] |
|  | Physicians dictate nurses’ practice | 3 | Barrier | All | ‘My supervising physician generally prefers to treat positive urine cultures in catheterized patients.’ [Tertiary Care; T3]  [Nurse:]‘…You can keep asking, ‘Can I pull the Foley?’ and they’ll [physicians] just say, ‘leave it in.’ [Secondary Care; S1] |
|  | Cultural norms regarding standard catheterisation practice for specific patient groups | 3 | Barrier | Pre-insertion, Insertion | ‘The biggest challenge was changing the culture of practice of inserting indwelling urinary catheter in all ICU patients’ [Secondary Care; S15] |
|  | Local champions | 2 | Facilitator | All | ‘Presence of an emergency physician champion to establish indications for urinary catheter placement resulted in fewer orders for catheters’ [Tertiary Care; T1] |
|  | TDF Domain: Social professional role and identity | | | | |
|  | Having a Hospital Epidemiologist in post | 2 | Facilitator | Removal | ‘Hospital epidemiologist in post was significantly and positively associated with: i) Urinary catheter reminder or stop-order/nurse-initiated catheter discontinuation’ [Secondary Care; S4] |
|  | Acceptance of responsibility for urinary catheterisation decision making | 2 | Barrier | Pre-insertion | ‘Nurses felt that the decision to maintain an indwelling catheter was up to the physician and did not consider it within their scope of practice.’ [Tertiary Care; T2] |
|  | CAUTI guidelines not perceived as relevant across Hospital departments | 1 | Barrier | All | ‘I looked at the criteria set forward indications for [urinary] catheter use. I think my gut reaction was that perhaps some of those were not as applicable in the Emergency Department setting… that maybe they were more devised for the inpatient setting.’ [Secondary Care; S1] |
|  | Nurses leading change in urinary catheterisation practice | 1 | Facilitator | All | ‘We’re really changing it [practice] from the bottom up, which I think is a great idea. We’re the ones doing the work so we’re the ones that make that change’ [Secondary Care; S7] |
| MOTIVATION (REFLECTIVE)  MOTIVATION (REFLECTIVE) | TDF Domain: Beliefs about consequences | | | | |
|  | Convenience and ease of monitoring | 5 | Mixed | All | ‘Catheters inserted for convenience purposes, for example, to accurately measure a patient’s urine output or to avoid frequent transfers to a bedpan or a bedside commode’ [Secondary Care; S18]  ‘Perception that urinary catheters are a potential source of harm not convenient and benign’ [Secondary Care; S7] |
|  | Perceived severity of CAUTI | 2 | Mixed | All | ‘Perception by nursing staff of urinary catheters and urinary tract infections as benign was quite common’ [Secondary care; S1]  ‘Perception that urinary catheters are a potential source of harm not convenient and benign’ [Secondary care’ S7] |
|  | Lack of perceived benefits to interventions targeting CAUTI | 2 | Barrier | All | ‘physicians did not realize the value or benefits of supporting program implementation’ [Care Homes; C1] |
|  | Improved patient hygiene | 1 | Facilitator | Pre-insertion | ‘you don’t want them lying in their own urine…It’s not a great for someone who’s wet with potential pressure areas and lying in their own wee (ED consultant physician).’ [Secondary Care; S17] |
|  | Pros and cons of reusable catheters | 1 | Mixed | Pre-insertion, Insertion | ‘Major concerns included the perceived increased risk of urinary tract infection and increased patient burden. Potential advantages included increased patient choice, cost savings and reducing the fear/likelihood of running out of catheters’ [Secondary Care; Community care; SC20] |
|  | TDF Domain: Beliefs about capabilities | | | | |
|  | Nurse empowerment | 1 | Facilitator | All | ‘Empowering nurses to identify and address CAUTI improvement opportunities: [We’re] really changing it [practice] from the bottom up, which I think is a great idea. We’re the ones doing the work so we’re the ones that make that change.’ [Secondary Care; S7] |
|  | Confidence in investigating and managing CAUTI | 1 | Facilitator | Post-insertion maintenance | ‘I feel confident in knowing when to order a urine culture on catheterised patients, …how to manage bacteriuria in catheterised patients…and that I can apply asymptomatic bacteriuria guidelines to my patients.’ [Tertiary Care; T3] |
|  | TDF domains: Goals | | | | |
|  | CAUTI is not a priority | 2 | Barrier | All | ‘… it’s difficult to find people that are excited about getting Foleys out of patients; other things take higher priority like central lines and VAP’ [Secondary Care; S1] |
