# Supplementary material for: Reducing catheter-associated urinary tract infections: a systematic review of barriers and facilitators and strategic behavioural analysis of interventions
Source: Implement Sci. 2020 Jul 6;15:44. doi: 10.1186/s13012-020-01001-2 (PMC7336619; doi:10.1186/s13012-020-01001-2)
Supplement: Supplementary file 11 — Additional file 11. Description of included nationally adopted interventions in England to reduce CAUTI [file 13012_2020_1001_MOESM11_ESM.docx]

# Additional file 11. Description of nationally implemented interventions to reduce CAUTI in the UK

| **Intervention Name** | **Intervention content*** | **Setting** |
| --- | --- | --- |
| NICE QS90: Urinary Tract Infections in Adults | Quality statement 2: Diagnosing urinary tract infections in adults with catheters  Quality statement: Healthcare professionals do not use dipstick testing to diagnose urinary tract infections in adults with urinary catheters.  Rationale: Dipstick testing is not an effective method for detecting urinary tract infections in catheterised adults. This is because there is no relationship between the level of pyuria and infection in people with indwelling catheters (the presence of the catheter invariably induces pyuria without the presence of infection). To ensure that urinary tract infections are diagnosed accurately and to avoid false positive results, dipstick testing should not be used.  Quality statement 5: Antibiotic treatment for asymptomatic adults with catheters and non-pregnant women  Quality statement: Healthcare professionals do not prescribe antibiotics to treat asymptomatic bacteriuria in adults with catheters and non-pregnant women.  Rationale: Antibiotics are not effective for treating asymptomatic bacteriuria in adults with catheters or non-pregnant women. Unnecessary treatment with antibiotics can also increase the resistance of bacteria that cause urinary tract infections, making antibiotics less effective for future use.  Quality statement 6: Antibiotic prophylaxis to prevent catheter-related urinary tract infection  Quality statement: Healthcare professionals do not prescribe antibiotic prophylaxis to adults with long-term indwelling catheters to prevent urinary tract infection unless there is a history of recurrent or severe urinary tract infection.  Rationale: Evidence shows that antibiotic prophylaxis is not effective in preventing symptomatic urinary tract infection in adults with long-term indwelling catheters unless there is a history of recurrent or severe urinary tract infection. | Community |
| NICE QS61: Infection prevention and control | Quality statement 4: Urinary catheters  Quality statement: People who need a urinary catheter have their risk of infection minimised by the completion of specified procedures necessary for the safe insertion and maintenance of the catheter and its removal as soon as it is no longer needed.  Rationale: Catheter-associated urinary tract infections comprise a large proportion of healthcare-associated infections, and can occur whether a person has either a short-term or a long-term catheter. There is a strong association between duration of urinary catheterisation and risk of infection, and catheters are sometimes inserted inappropriately or there is a delay in removing them. This risk is greatly reduced by complying with all parts of the process for safe catheter insertion, maintenance and removal as soon as it is no longer needed. This is important in terms of both infection prevention and patient comfort and experience. | Primary, Community, Secondary |
| The Health and Social Care Act 2008 Code of Practice on the prevention and control of infections and related guidance | Criterion 9: Have and adhere to policies, designed for the individual’s care and provider organisations that will help to prevent and control infections.  a. Standard infection prevention and control precautions  All staff should have training on hand hygiene and when and how personal protective equipment should be used. Policies should also be in place for the safe handling and disposal of sharps and the safe disposal of waste.  6.3 Where staff undertake procedures, which require skills such as aseptic technique, staff must be trained and demonstrate proficiency before being allowed to undertake these procedures independently  s. Use and care of invasive devices  Policy should be based on evidence-based guidelines and should be easily accessible by all relevant care workers. Compliance with policy should be audited. Information on policy should be included in infection prevention and control training programmes for all relevant staff groups. | Primary, Community, Secondary |
| Department of Health and Public Health England (2013) Prevention and control of infections in care homes: an informative resource | Use and care of invasive devices Invasive devices such as a urinary catheter or intravenous line will increase a resident’s risk of acquiring an infection and the care home should have a policy in place for the care of invasive devices, the policy should be audited on a planned basis. Where residents have an invasive device in place, this should be fully documented in the care plan and the resident should be monitored for signs of infection. Staff should be trained in the care of residents with invasive devices and in how to recognise signs and symptoms of infection.  Urinary catheter care: Residents with a urinary catheter in place are at an increased risk of acquiring an infection. Bacteria can enter the urethra at the point where the catheter enters the body. The date of catheter insertion and the indication for catheterisation should be recorded in the resident’s notes. The resident's clinical need for catheterisation should be reviewed regularly by the GP or district nurse and the urinary catheter removed as soon as possible. The following advice will minimise the risk of the resident acquiring an infection.  Handling the catheter  • Hands should be washed and a clean pair of non-sterile gloves should be put on before handling the catheter or drainage bag. Hands should be cleaned again after removing gloves.  • The point at which the catheter enters the body should be cleaned daily with soap and water.  Managing the drainage system  • The drainage bag or catheter valve should be connected to the catheter at all times, except when changing the bag. This ‘closed system’ reduces the risk of infection.  • At night, the special night drainage bag should be added without breaking the closed system.  • The drainage bag should be kept lower than the bladder to allow urine to drain.  • The bag should not be allowed to touch the floor because this can increase the infection risk. Catheter bag stands should be used.  • The drainage bag should be emptied regularly to maintain the flow of urine. | Nursing Homes |
| Safety thermometer | The tool is built around 4 steps:  Step 1: Setting an improvement goal  Step 2: Ongoing monitoring  Step 3: Identifying and reviewing special cause  Step 4: Working out the CQUIN payment | Primary, Community, Secondary, Nursing homes |
| Epic 3 | Assessing the need for catheterisation  UC1 Only use a short-term indwelling urethral catheter in patients for whom it is clinically indicated, following assessment of alternative methods and discussion with the patient.  UC2 Document the clinical indication(s) for catheterisation, date of insertion, expected duration, type of catheter and drainage system, and planned date of removal.  UC3 Assess and record the reasons for catheterisation every day. Remove the catheter when no longer clinically indicated.  Selection of catheter type  UC4 Assess patient’s needs prior to catheterisation in terms of:  • latex allergy  • length of catheter (standard, female, paediatric)  • type of sterile drainage bag and sampling port (urometer, 2-L bag, leg bag) or catheter valve  • comfort and dignity.  UC5 Select a catheter that minimises urethral trauma, irritation and patient discomfort, and is appropriate for the anticipated duration of catheterisation.  UC6 Select the smallest gauge catheter that will allow urinary outflow and use a 10-mL retention balloon in adults (follow manufacturer’s instructions for paediatric catheters). Urological patients may require larger gauge sizes and balloons.  Catheter insertion  UC7 Catheterisation is an aseptic procedure and should only be undertaken by healthcare workers trained and competent in this procedure.  UC8 Clean the urethral meatus with sterile, normal saline prior to the insertion of the catheter.  UC9 Use lubricant from a sterile single use container to minimise urethral discomfort, trauma and the risk of infection. Ensure the catheter is secured comfortably.  Catheter maintenance  UC10 Connect a short-term indwelling urethral catheter to a sterile closed urinary drainage system with a sampling port.  UC11 Do not break the connection between the catheter and the urinary drainage system unless clinically indicated.  UC12 Change short-term indwelling urethral catheters and/or drainage bags when clinically indicated and in line with the manufacturer’s recommendations.  UC13 Decontaminate hands and wear a new pair of clean non-sterile gloves before manipulating each patient’s catheter. Decontaminate hands immediately following the removal of gloves.  UC14 Use the sampling port and the aseptic technique to obtain a catheter sample of urine.  UC15 Position the urinary drainage bag below the level of the bladder on a stand that prevents contact with the floor.  UC16 Do not allow the urinary drainage bag to fill beyond three-quarters full.  UC17 Use a separate, clean container for each patient and avoid contact between the urinary drainage tap and the container when emptying the drainage bag.  UC18 Do not add antiseptic or antimicrobial solutions to urinary drainage bags.  UC19 Routine daily personal hygiene is all that is required for meatal cleansing.  Education of patients, relatives and healthcare workers  UC20 Do not use bladder maintenance solutions to prevent catheter-associated urinary tract infection.  UC21 Healthcare workers should be trained and competent in the appropriate use, selection, insertion, maintenance and removal of short-term indwelling urethral catheters.  UC22 Ensure patients, relatives and carers are given information regarding the reason for the catheter and the plan for review and removal. If discharged with a catheter, the patient should be given written information and shown how to:  • manage the catheter and drainage system  • minimise the risk of urinary tract infection  • obtain additional supplies suitable for individual needs.  System interventions for reducing the risk of infection  UC23 Use quality improvement systems to support the appropriate use and management of short-term urethral catheters and ensure their timely removal. These may include:  • protocols for catheter insertion  • use of bladder ultrasound scanners to assess and manage urinary retention  • reminders to review the continuing use or prompt the removal of catheters  • audit and feedback of compliance with practice guidelines  • continuing professional education.  UC24 No patient should be discharged or transferred with a short-term indwelling urethral catheter without a plan documenting the:  • reason for the catheter  • clinical indications for continuing catheterisation  • date for removal or review by an appropriate clinician overseeing their care. | Secondary |
| High Impact Intervention for best practice insertion and care | Using the high impact interventions tool  1. Each time a care element is performed, insert a [tick] in the relevant column. If the action is not performed leave it blank.  2. Ensure you only [tick] it when an element of care is performed correctly or if the element is not applicable.  3. Calculate the totals and compliance levels by totalling the columns and using the tools provided.  4. Your goal is to perform every element of care every time it is needed. The ""All elements performed"" column should be ü when every care element is given correctly. This should total to 100% compliance when all care elements have been given correctly on every occasion.  5. Where elements have not been performed overall compliance will be less than 100%. This provides immediate feedback for users of the tool on those elements missed, and actions can then be taken to improve on compliance levels.  6. The percentage compliance figures for individual care elements show you where you need to focus effort to improve overall compliance.  7. The number of times when all elements are performed should be the same as the number of observations you perform. For example if you monitor the care process 10 times, then there should be 10 occasions when all elements were performed.  High Impact Interventions to prevent catheter associated urinary tract infection  Aim  To reduce the incidence and consequences of urinary tract infection associated with both short and long term urethral catheters.  Why use the high impact intervention?  Catheter associated urinary tract infections comprise a large proportion of healthcare associated infections and occur whether a person has either a short term catheter or long term catheter. There is a strong association between duration of urinary catheterisation and risk of infection and these are becoming more serious with the continued development of a wide range of multi-resistant bacteria which can cause catheter associated urinary tract infections and associated life threatening complications (RCN 2012). Risks are greatly reduced complying with all parts of the process for safe catheterisation, maintenance, and removal as soon as no longer needed. This is important in both terms of promoting comfort, safety and infection prevention control measures. (NICE guidelines 2014).  Elements of the care process  There are two sets of actions outlined below as good practice.  a. Insertion phase  b. Routine maintenance and assessment for continuing indication phase  Insertion phase  1. Assessment for catheter indication  Assessment of the need of the catheter is to be documented ensuring a clear clinical indication which includes exploring alternative options.  2. Aseptic procedure  Catheterisation should follow an aseptic procedure including hand hygiene and is documented.  3. Urethral meatus  The meatus should be cleaned with normal saline prior to insertion. Use a lubricant gel from a sterile single use sachet/syringe to minimise urethral trauma.  4. Catheter insertion documentation  Document as a minimum the following:  • date of insertion,  • indication for catheterisation  • catheter size  • type of catheter and planned date for removal.  Routine maintenance and assessment for continuing indication phase  1. Hand hygiene  Hands are decontaminated immediately before and after each episode of patient contact using the correct hand hygiene technique.  2. Personal protective equipment  Wear personal protective equipment only when indicated and in accordance with local policy.  3. Assessment  Daily assessment of the need of the short term urinary catheter needs to be clearly documented. Long term catheters should be reviewed regularly, at least every catheter change and documented.  4. Catheter hygiene  Routine daily personal hygiene is required for meatal cleaning.  5. Routine maintenance  • Do not break the connection between the catheter and the urinary drainage system unless clinically indicated. Use a separate clean/disposable container when emptying the drainage bag.  • Document on the drainage bag when last changed and should be changed in line with the manufacturer’s recommendation. The urinary catheter tubing and leg bag should be fixed to the patient’s leg using a leg strap.  6. Patient information  Ensure patients and carers are given information regarding the reason for the catheter and the plan for review and removal e.g. indwelling urinary catheter passport. | Secondary, Community |
| Catheter Care: Royal College of Nursing Guidance for nurses | You need to apply:   - legislation, policy and good practice, the current international, European, UK and national legislation, guidelines and local policies, protocols and procedures which affect your work practice in relation to the care of individuals using urinary catheters - a factual knowledge of the current European and national legislation, national guidelines, organisational policies and protocols in accordance with clinical/corporate governance which affect your work practice in relation to the care of individuals using urinary catheters (not sure if this is sufficient to code as education).   Practice recommendations  The suggested structure for gaining competence in catheterisation is as follows:   - gain a theoretical knowledge and understanding in aspects of catheterisation - observe model/manikin being catheterised - practise catheterisation on a model/manikin under supervision until confident - observe catheterisation performed by others on actual patients - undertake supervised catheterisation on actual patients [does not meet definition of behavioural practice/rehearsal] - be able to catheterise without direct supervision - gain experience and become confident - become a competent mentor for others - have catheterisation technique observed as part of clinical audit (Saving lives).   In all care settings nurses should have observed clinical practice for the following procedures:  assessing individual patients to ensure catheterisation is still required   - obtaining a CSU - changing urinary drainage systems - emptying a urine bag or catheter valve - catheter insertion - catheter removal - meatal cleansing - bag position and support. - In relation to all aspects of catheter care it is recommended that nurses have a formal update at least every five years, and more often if appropriate or required.   Documentation  What you need to do:   - you need to record clearly, accurately, and correctly any relevant information in the ongoing catheter care records. You also need to be aware of the importance of documentation, the data protection act, care of patient records and disclosure of information with consent from the patient and your employer and the legal and professional consequences of poor practice.   Some general principles relating to documentation apply. These include confidentiality, legibility and that documents can be photocopied. Documentation has a number of purposes, and these include:   - contributing to and establishing a diagnosis - influencing a care bundle and pathway of catheter care for an individual patient - a legal record of care bundle provision and what actually happened - effective communication for other health care professionals involved in a patient’s care - a point of reference used to influence decisions for further interventions - facilitating product tracing, if for any reason an individual patient experiences product failure - a record for the investigation of complaints and/or litigation - facilitating critical reflective thinking - focus for clinical professional supervision and identification of learning needs - completing an episode of care, end of a procedure or care bundle (group of procedures, tasks or activities forming a bundle of care).   In the development of documentation related to catheter care, ensure the documentation is designed to be audit friendly and understood by the patient. Regard must be given to the documentation of consent, whether this is written or verbally given.  Catheter insertion documentation should include:   - the reason for the catheterisation, catheter change or ongoing need for a catheter with all its risks - well/unwell/serious health status of the patient prior to catheterisation - is the patient febrile, temperature (over 39°C, are blood cultures needed)? - if taking antibiotics for a urinary tract infection, are they effective? - is the individual patient in any form of localised discomfort or pain? - initially it may be necessary to record fluid intake balanced against urinary output and in some cases this may be ongoing (e.g. renal function and or failure) - the results of a risk assessment prior to catheterisation - allergy status (for example latex, gels and medication) - consent obtained for the procedure; some organisations now require this to be in written form - if antibiotic cover was used, state drug and dosage (see infection control and catheter care section on page 43) - meatal or genital abnormalities observed, including discharge - if the insertion was easy or difficult - indications used to ensure catheter was inserted correctly (in men – amount of catheter inserted, obstruction felt at prostatic area, patient reaction to passing the prostatic area, urine drained, no resistance to balloon inflation, no patient reaction or pain related to balloon inflation, free movement of the catheter once balloon inflated) - if urine is drained, the amount, colour, smell and, if necessary, dipstick and record the result (blood, protein, pH, glucose, nitrite, leucocytes) - if no urine drains, document what actions you took - brand, catheter name, material, tip type, catheter length, charriére size, balloon size, batch number, expiry date - cleaning fluid used - lubricant/anaesthetic gel used - if specimens were sent, and why.   Drainage equipment documentation should include:   - is this type of urinary drainage system appropriate for this particular patient? - appropriateness of brand, capacity, tube length - support system being used and if it was appropriate - if a link system is being used and the type of night bag (single use or drainable) - check when the drainage system was previously changed and if this was appropriate. Note date of change of bag or valve - urinary drainage bags are dated whenever they are changed within health and social care settings - batch number of equipment and sterility expiry date - any problems with product function - any problems with the supply of equipment - any problems with comfort - any associated skin or allergy problems - any problems related to lifestyle or daily activities - is the system being used cost effective?   Catheter removal documentation should include:   - that the length of time the catheter was in-situ was appropriate for the type being used - the type of catheter, drainage system and support garments/straps being removed were appropriate - the catheter tip and balloon were intact upon removal - if encrustation was present, and to what degree - if the section of the catheter retained within the bladder was clean or dirty or if debris was evident - if the balloon deflated appropriately - if the catheter was removed because of blockage, the catheter was not present to allow direct observation, was it dissected to identify the cause and severity? - if the removal was painful - if blood was present, and if so, where (catheter tip, in the bag, around the meatus, clots in drainage bag tube) and to what degree (clot, red coloured urine, - meatal bleeding, frank haematuria)? - observation around the meatus for any abnormalities (inflammation, swelling, meatal erosion, discharge/amount/colour) - observations of urine for signs of infection (cloudy, debris, amount, colour and smell) - patient tolerance of the catheter.   Ongoing observations documentation should include, if a problem occurs:   - the health status of the patient (well/unwell/seriously ill) - is the patient febrile, temperature (over 39°C, are blood cultures needed)? - if taking antibiotics for a urinary tract infection, type, duration of course and are they effective? - patient tolerance of the catheter and associated drainage system - is the individual patient in any form of discomfort or pain? - the fluid intake balanced against urinary output - if first-time catheterisation takes place in primary care, it is safe practice to monitor urine output for four hours after catheterisation. If the patient passes more than 200mls per hour after initial drainage, they need to be referred to the accident and emergency unit for fluid replacement as they are in risk of hypovolaemic shock - hourly urine output in critically ill patients - bowel activity - renal status - blood results (prostatic specific antigen PSA, urea, creatinine), the results, diagnosis and further interventions - glycosuria, in a known diabetic is indicative of poor control and infection risk, if diagnosis is unknown then further investigations are needed to establish a diagnosis - blood pressure status, in relation to proteinuria and nocturnal polyuria (increased night time urinary output) to help establish a diagnosis - immune status influencing interventions and associated risks - communication with other members of the multidisciplinary team regarding this individual patient’s observations.   Anatomy and physiology  What you need to know:  You need to apply:   - an indepth understanding of the anatomy and physiology of the male and female lower urinary tract in relation to lower urinary tract function and continence status including:   a) urine production and what influences this  b) normal micturition  c) the nervous system including autonomic dyssreflexia  d) the bowel and its links to voiding problems  e) endocrine system  f ) sexual function and links to catheter usage  g) the prostate gland, urethral sphincters and the urethra  h) anatomy and physiology applied to voiding dysfunction and how a urethral urinary catheter could be used to relieve this (BCT 5.1)  i) anatomy and physiology links of how common catheter related complications occur.   - an indepth understanding of how to educate and advise individuals in the use of catheters in relation to their anatomy, its function and sensation.   Consent  What you need to do:   - you need to obtain the individual’s valid consent for the procedure (catheterisation), and, in terms of the care and support of the individual, know how to obtain valid consent and how to confirm that sufficient information has been provided on which to base this judgement (catheterisation). - avoid coercing or restraining patients for catheterisation, including aspects of ongoing catheter care, as this is assault in law and demonstrates a lack of consent.   Reasons for, and decisions influencing, catheterisation  What you need to do   - During individual assessment when instrumental bladder drainage is deemed necessary, consideration needs to be given to the patient’s suitability for intermittent, suprapubic or urethral catheterisation. - You need to understand the reasons for catheterisation and constantly review the need for continued catheter usage. - Where it is viewed as appropriate for the patient to use a catheter, such as end-of-life care, disability, unfit for surgery, nurses must remember that the risks associated with catheter usage are of a serious nature that increasingly may become more difficult to justify. - Never catheterise or continue catheter usage for nursing convenience. - Nurses must ensure that catheterisation is based on a balanced decision with more benefits than disadvantages, in consultation with the patient, where possible. - Routine catheterisation must not be routinely supported by nurses, particularly in specific patient groups such as fractured neck or femur. - Incontinence is rated as a major factor in the development of pressure ulcers. Inserting an indwelling catheter could be assessed as reducing this risk, however with a catheter in-situ, there is less need for the patient to mobilise as they would with toileting or pad changes, so the risk can be higher (BCT 5.1). - Catheterisation of patients who are agitated and/or cognitively impaired is best avoided where possible. - Where a residual volume of urine is identified, the patient’s symptom and severity profile along with their renal function and cognitive status must be considered prior to considering catheterisation. - Where a residual volume of urine is identified and a decision to catheterise is made, it is imperative that the nurse ensures that the route of catheterisation is made within a multi-disciplinary team (MDT) framework. - Nurses must always assess clinical need for catheter usage as part of their professional role, even if medical directives state ‘to catheterise’. - When an indwelling catheter is inserted the nurse should consider and plan for early removal as the infection risk increases on a daily basis. - Nurses should not, under any circumstance, present or promote catheterisation to patients as an easy, best option to regain continence. - With the continued development of multi-resistant bacteria and lack of effective antibiotics, nurses must be mindful of the serious implications when making the decision to catheterise.   Suprapubic catheterisation  Positive aspects of suprapubic catheterisation:   - there is no risk of urethral trauma, necrosis or catheter induced urethritis - there is greater comfort, particularly for patients who are wheelchair users (these are two of six pros of suprapubic catheterisation)   Negative aspects of suprapubic catheterisation:   - altered body image (Addison and Mould, 2000) - cystostomy complications (Ichsan and Hunt, 1987), including swelling, infection, cellulitis, encrustation and granulation may be encountered (Addison, 1999c) *(these are two of 11 cons of suprapubic catheterisation)*   Trial without catheter  How to minimise discomfort during a TWOC:   - in removing a catheter at the start of a TWOC, check water volume in balloon. Avoid pulling on the syringe as this may create a vacuum and cause the balloon to cuff making removal difficult. Instead allow water to drain out of the balloon under its own pressure.   Where to perform a TWOC and why:   - at home, if possible, as it is more relaxed for the patient and reduces the risk of cross infection by not bringing them back into a hospital ward environment   Intermittent self-catheterisation   - before commencing a patient on intermittent catheterisation, their symptom severity profile, renal function, risk assessment, psychological and physical ability to perform intermittent catheterisation and residual urine status must be considered. It is not best practice to initiate intermittent catheterisation based solely on the residual urine status | Primary, Community, Secondary, Nursing homes |
| HOUDINI Protocol | HOUDINI is an acronym used to list the indications for continued use of a UC:   - Haematuria - Obstruction - Urology surgery - Decubitus ulcer - Input and output measurement - Nursing end of life care - Immobility   If none of the above criteria are met, the registered nurse is to discontinue urinary catheter per nursing protocol and document in CERNER.  After urinary catheter removal   - assess for voiding within six (6) hours of removal - if patient spontaneously voids within six (6) hours but it's <200ml, perform bladder scan initiate straight catheterisation if PVR is >200ml. - if patient spontaneously voids within six (6) hours, but is incontinent, perform bladder scan, straight catheterisation if PVR is >200ml. - If patient has not voided within six (6) hours after straight catheterisation, notify physician. | Secondary Care |
| To Dip or Not to Dip | Content coded from You Tube film. Relevant coded content is summarised here:  BCT 5.1 (Information about health consequences)- Information about the link between catheter use and UTI and the link between antibiotics and side-effects  BCT 9.1(Credible source) - Cites NICE guidance on catheter care  BCT 2.2 (Feedback on behaviour)- Feedback on antibiotic prescribing and urine dip usage  BCT 2.3 (Self-monitoring of behaviour) and BCT 2.5 (Monitoring of outcome(s) of behaviour without feedback) - Assessment tool to record behaviour of nursing home staff and symptoms (outcomes of patients)  BCT 4.1 (Instruction on how to perform the behaviour) - Instruction on how to complete the assessment tool  BCT 8.1 (Behavioural practice/rehearsal) - Practice dehydration management  BCT 10.10 (Reward (outcome) - Certificate at the end of the module | Nursing homes |
| Catheter Passport | All service users with a urinary catheter are at increased risk of acquiring a UTI and the longer a catheter is in place the greater the risk.  Catheter hygiene  Routine personal hygiene, such as a daily bath or shower, is important to maintain catheter hygiene. For those who are unable to bathe or shower, staff should wash the genital area including around the catheter at least daily with soap and water. For women, it is important to wash the genital area from front to back to prevent contamination from the anal area.  Always remember that catheter straps should be used to secure the catheter tube to the leg to prevent trauma  Emptying a catheter bag  A catheter drainage bag should not be emptied more often than necessary as this increases the risk of infection. However, the bag must be emptied when it is no more than two thirds full to avoid back flow of urine into the bladder. There is an increased risk of acquiring an infection when the catheter bag is emptied. Therefore, it is essential to follow good practice.   - Before emptying the bag, always wash hands and wear a disposable apron and gloves. - In a healthcare setting, the tap on the drainage bag should be wiped with an alcohol wipe before opening the tap and after closure, to reduce the risk of transmission of infection. In a person’s own home, use a clean tissue to wipe the tap after closure to prevent drips. - Empty the urine into a container by opening the drainage tap. - In a healthcare setting, the container should be single use and disposable or if reusable, washed in an automated bed pan washer after each use. In a person’s own home, the container can be used again after washing with detergent and warm water and dried with a disposable paper towel, such as kitchen roll. Avoid contact between the tap on the drainage bag and the container to prevent contamination and infection. - Urine should be disposed of into a sluice or toilet. - Remove gloves and disposable apron and wash hands with warm water and liquid soap.   Changing a catheter bag  Catheter bags, including leg bags, should be sterile and changed according to the manufacturer’s instructions, usually weekly. There is an increased risk of acquiring an infection when the catheter bag is changed. Therefore, it is essential to follow good practice.   - Before changing the bag, always wash hands and wear a disposable apron and gloves. - When detaching the used bag from the catheter, to prevent contamination and infection, do not touch the end of the catheter. - When removing the cap from the new catheter bag tube, to prevent contamination and infection, do not touch the end of the tube. - Empty the urine from the bag into a sluice or toilet and dispose of the bag as non-infectious/offensive waste. In a person’s own home, double wrap the bag and dispose of as household waste. - Remove gloves and disposable apron and wash hands with liquid soap and warm water. - Always record when the catheter bag is changed.   Overnight drainage bags  If a leg bag is used during the day, an additional larger linked drainage bag (night bag) should be used for overnight use. The night bag should be attached to the leg bag to keep the original system intact.   - Always wash hands and wear a disposable apron and gloves when attaching a night bag. - Attach the night bag to a stand to prevent the tap from touching the floor. - In a healthcare setting, wipe the leg bag drainage tap with an alcohol wipe to reduce the risk of transmission of infection. In a person’s own home, it is not necessary to wipe the tap. - When removing the cap from the new night bag tube, to prevent contamination, do not touch the end before attaching it to the drainage tap on the leg bag. - Remove gloves and disposable apron and wash hands. - Night bags are single use only and should be disposed of on removal and should not be used again. | Primary, Community, Nursing homes |
| NICE catheter audit tools | All catheterisations carried out by healthcare workers should be aseptic procedures. After training, healthcare workers should be assessed for their competence to carry out these types of procedures. When changing catheters in patients with a long-term indwelling urinary catheter:   - do not offer antibiotic prophylaxis routinely - consider antibiotic prophylaxis for patients who have a history of symptomatic urinary tract infection after catheter change or experience trauma] during catheterisation   Long-term urinary catheters  Education of patients, their carers and healthcare workers   - Patients and carers should be educated about and trained in techniques of hand decontamination, insertion of intermittent catheters where applicable, and catheter management before discharge from hospital. - Community and primary healthcare workers must be trained in catheter insertion, including suprapubic catheter replacement and catheter maintenance. [2003] - Follow-up training and ongoing support of patients and carers should be available for the duration of long-term catheterisation.   Assessing the need for catheterisation   - Indwelling urinary catheters should be used only after alternative methods of management have been considered. - The patient's clinical need for catheterisation should be reviewed regularly and the urinary catheter removed as soon as possible. - Catheter insertion, changes and care should be documented.   Catheter drainage options   - Following assessment, the best approach to catheterisation that takes account of clinical need, anticipated duration of catheterisation, patient preference and risk of infection should be selected. - Intermittent catheterisation should be used in preference to an indwelling catheter if it is clinically appropriate and a practical option for the patient. - Offer a choice of either single-use hydrophilic or gel reservoir catheters for intermittent self-catheterisation. - Select the type and gauge of an indwelling urinary catheter based on an assessment of the patient's individual characteristics, including: age any allergy or sensitivity to catheter materials gender history of symptomatic urinary tract infection patient preference and comfort previous catheter history reason for catheterisation. - In general, the catheter balloon should be inflated with 10 ml of sterile water in adults and 3–5ml in children. - In patients for whom it is appropriate, a catheter valve may be used as an alternative to a drainage bag (BCT 4.1).   Catheter insertion   - All catheterisations carried out by healthcare workers should be aseptic procedures. After training, healthcare workers should be assessed for their competence to carry out these types of procedures. - Intermittent self-catheterisation is a clean procedure. A lubricant for single patient use is required for non-lubricated catheters. - For urethral catheterisation, the meatus should be cleaned before insertion of the catheter, in accordance with local guidelines/policy. - An appropriate lubricant from a single-use container should be used during catheter insertion to minimise urethral trauma and infection.   Catheter maintenance   - Indwelling catheters should be connected to a sterile closed urinary drainage system or catheter valve. - Healthcare workers should ensure that the connection between the catheter and the urinary drainage system is not broken except for good clinical reasons (for example changing the bag in line with the manufacturer's recommendations). - Healthcare workers must decontaminate their hands and wear a new pair of clean, non-sterile gloves before manipulating a patient's catheter, and must decontaminate their hands after removing gloves. - Patients managing their own catheters, and their carers, must be educated about the need for hand decontamination before and after manipulation of the catheter, in accordance with the recommendations in the standard principles section. - Urine samples must be obtained from a sampling port using an aseptic technique. - Urinary drainage bags should be positioned below the level of the bladder, and should not be in contact with the floor. - A link system should be used to facilitate overnight drainage, to keep the original system intact. - The urinary drainage bag should be emptied frequently enough to maintain urine flow and prevent reflux, and should be changed when clinically indicated. - The meatus should be washed daily with soap and water. - To minimise the risk of blockages, encrustations and catheter-associated infections for patients with a long-term indwelling urinary catheter: - develop a patient-specific care regimen consider approaches such as reviewing the frequency of planned catheter changes and increasing fluid intake document catheter blockages. - Bladder instillations or washouts must not be used to prevent catheter associated infections. - Catheters should be changed only when clinically necessary or according to the manufacturer's current recommendations. - When changing catheters in patients with a long-term indwelling urinary catheter: do not offer antibiotic prophylaxis routinely consider antibiotic prophylaxis for patients who: have a history of symptomatic urinary tract infection after catheter change or experience trauma during catheterisation. | Primary, Community |
| Only text relevant to health professional behaviour change for CAUTI-related behaviours is included here | | |
