# Supplementary material for: Reducing catheter-associated urinary tract infections: a systematic review of barriers and facilitators and strategic behavioural analysis of interventions
Source: Implement Sci. 2020 Jul 6;15:44. doi: 10.1186/s13012-020-01001-2 (PMC7336619; doi:10.1186/s13012-020-01001-2)
Supplement: Supplementary file 12 — Additional file 12. BCTs, intervention functions and policy categories identified in each intervention [file 13012_2020_1001_MOESM12_ESM.docx]

**Additional file 12. BCTs, intervention functions and policy categories identified in each intervention**

| **Intervention Name** | **Settings** | | **Intervention functions** | | **Policy Categories** | | **Behaviour Change Techniques** | | **Behavioural phase** | | | **Target group** |
| --- | --- | --- | --- | --- | --- | --- | --- | --- | --- | --- | --- | --- |
| The Health and Social Care Act 2008 Code of Practice on the prevention and control of infections and related guidance | Primary, Community, Secondary | | Education, Training | | Legislation | | - Instruction on how to perform the behaviour - Monitoring of behaviours by others without feedback - Behavioural practice/ rehearsal | | All | | | Nurse, secondary care physician, GP |
| NICE QS90: Urinary Tract Infections in Adults | Community | | Education | | Guidelines | | - Instruction on how to perform the behaviour - Information about health consequences - Goal setting (behaviour) | | Pre-insertion, post-insertion maintenance | | | Nurse, GP |
| NICE QSG1: Infection prevention and control | Primary, Community, Secondary | | Education | | Guidelines | | - Instruction on how to perform the behaviour - Information about health consequences - Goal setting (behaviour) | | Insertion, post-insertion maintenance | | | Nurse, secondary care physician, GP |
| NICE catheter audit tools | Primary, Community | | Education, Enablement | | Guidelines | | - Goal setting (behaviour) - Self-monitoring (behaviour) - Self-monitoring (outcomes) - Action planning - Instruction on how to perform the behaviour - Credible source - Review behavioural goals - Social support (practical) - Discrepancy between behaviour and goal - Information about health consequences - Information about social environmental consequences | | Insertion, post-insertion maintenance | | | Nurse, secondary care physician, GP |
| Department of Health and Public Health England (2013) Prevention and control of infections in care homes: an informative resource | Nursing Homes | | Education, Training | | Guidelines | | - Instruction on how to perform the behaviour - Information about health consequences - Monitoring of outcome of behaviour without feedback - Monitoring of behaviours by others without feedback | | Insertion, post-insertion maintenance | | | Care home staff |
| Safety thermometer | Primary, Community, Secondary, Nursing homes | | Education, Enablement, Incentivisation | | Service provision | | - Goal-setting (outcome) - Self-monitoring (behaviour) - Self-monitoring (outcome) - Feedback on behaviour - Feedback on outcome - Reward (outcome) - Information Social environmental consequences - Credible Source - Social comparison | | Post-insertion maintenance | | | All |
| Epic 3 | Secondary | | Education, Enablement | | Guidelines | | - Self-monitoring (behaviour) - Information about health consequences - Instruction on how to perform the behaviour - Social support (practical) - Prompts/cues - Feedback (behaviour) - Feedback (outcomes) | | Insertion, post-insertion maintenance, removal | | | Secondary care nurses and physicians |
| High Impact Intervention for best practice insertion and care | Secondary, Community | | Education, Training, Enablement | | Guidelines | | - Self-monitoring (behaviour) - Credible source - Information social environmental consequences - Goal-setting (behaviour) - Discrepancy between behaviour and goal - Feedback (behaviour) - Feedback (outcome) - Information about health consequences | | Insertion, post-insertion maintenance | | | Nurse, secondary care physician, GP |
| Catheter Care: Royal College of Nursing Guidance for nurses | Primary, Community, Secondary, Nursing homes | | Education, training, modelling, enablement | | Guidelines | | - Credible source - Social support (practical) - Self-monitoring (behaviour) - Self-monitoring (outcome) - Instruction on how to perform the behaviour - Demonstration of the behaviour - Behavioural practice/ rehearsal - Identification of self as a role model - Information on health consequences - Information on emotional consequences - Information on social/ environmental consequences - Monitoring of behaviour by others without feedback | | All | | | Nurse |
| **Locally initiated widely adopted interventions** | | | | | | | | | | | | |
| HOUDINI Protocol | | Secondary Care | | Education, Enablement | | Guidelines | | - Instruction on how to perform the behaviour - Restructuring the social environment | | Removal | Secondary care nurses and physicians | |
| Catheter Passport | Primary, Community, Nursing homes | | Education, Enablement, Modelling | | Guidelines | | - Information about health consequences - Social support (practical) - Instruction on how to perform the behaviour - Demonstration of the behaviour | | Post-insertion maintenance | | | All |
