# Supplementary material for: Reducing catheter-associated urinary tract infections: a systematic review of barriers and facilitators and strategic behavioural analysis of interventions
Source: Implement Sci. 2020 Jul 6;15:44. doi: 10.1186/s13012-020-01001-2 (PMC7336619; doi:10.1186/s13012-020-01001-2)
Supplement: Supplementary file 13 — Additional file 13. Examples of BCT identification [file 13012_2020_1001_MOESM13_ESM.docx]

**Additional file 13. Examples of BCT identification**

| **Behaviour Change Technique label** | **Definition from Taxonomy v1*** | **Example from analysed interventions [Intervention label]** |
| --- | --- | --- |
| Goal-setting (behaviour) | Set or agree on a goal defined in terms of the behaviour to be achieved | The audit standards in this document include a reference to the guideline recommendation numbers, and any associated NICE quality standard statements and exceptions. NICE recommends compliance of 100%. e.g. ‘Healthcare workers must decontaminate their hands and wear a new pair of clean, non-sterile gloves before manipulating a patient’s catheter, and must decontaminate their hands after removing gloves. [NICE Catheter Audit Tool] |
| Goal-setting (outcome) | Set or agree on a goal defined in terms of a positive outcome of wanted behaviour. Note code as outcome if goal unspecified or if a behavioural outcome. | Step 1: Setting an improvement goal’ [Safety Thermometer] |
| Review behaviour goal(s) | Review behaviour goal(s) jointly with the person and consider modifying goal(s) or behaviour change strategy in light of achievement. This may lead to re-setting the same goal, a small change in that goal or setting a new goal instead of (or in addition to) the first, or no change | ‘NICE recommends compliance of 100%. If this is not achievable an interim local target could be set, although 100% should remain the ultimate aim’ [NICE Catheter Audit Tool] |
| Discrepancy between current behaviour and goal(s) | Draw attention to discrepancies between a person’s current behaviour (in terms of the form, frequency, duration, or intensity of that behaviour) and the person’s previously set outcome goals, behavioural goals or action plans (goes beyond self-monitoring of behaviour) | Your goal is to perform every element of care every time it is needed (. The "All elements performed" should total to 100% compliance when all care elements have been given correctly on every occasion. Where elements have not been performed overall compliance will be less than 100%. [High Impact Intervention for best practice insertion and care] |
| Action planning | Prompt detailed planning of performance of the behaviour (must include at least one of context, frequency, duration and intensity). Context may be environmental (physical or social) or internal (physical, emotional or cognitive) (includes ‘Implementation Intentions’) | Action planning template:  **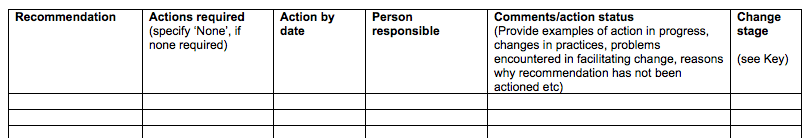**  [NICE Catheter Audit Tool] |
| Monitoring of outcome of behaviour by others without feedback | Observe or record behaviour with the person’s knowledge as part of a behaviour change strategy | ‘Where residents have an invasive device in place, this should be fully documented in the care plan and the resident should be monitored for signs of infection’ [Department of Health & Public Health England (2013) Prevention and control of infections in care homes: an informative resource] |
| Monitoring of the behaviour by others without feedback | Observe or record outcomes of behaviour with the person’s knowledge as part of a behaviour change strategy | ‘Compliance with policy should be audited’ [The Health and Social Care Act 2008 Code of Practice on the prevention and control of infections and related guidance] |
| Feedback (on outcome of behaviour)  (on outcome of behaviour) | Monitor and provide feedback on the outcome of performance of the behaviour | Dashboard reporting rates of catheterisation (behaviour) and UTI (outcome of behaviour)overtime:  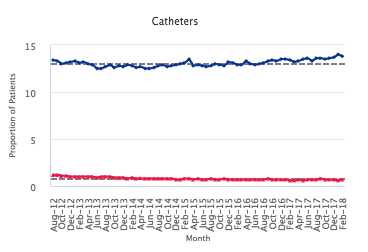  [Safety Thermometer] |
| Self-monitoring (behaviour) | Establish a method for the person to monitor and record their behaviour(s) as part of a behaviour change strategy | ‘Each time a care element is performed, insert a [tick] in the relevant column. If the action is not performed leave it blank. Ensure you only [tick] it when an element of care is performed correctly or if the element is not applicable.’ [High Impact Intervention for best practice insertion and care] |
| Self-monitoring (outcomes behaviour) | Establish a method for the person to monitor and record the outcome(s) of their behaviour as part of a behaviour change strategy | ‘Ongoing observations documentation should include, if a problem occurs: the health status of the patient (well/unwell/seriously ill), if the patient febrile, temperature (over 39°C, are blood cultures needed), if taking antibiotics for a urinary tract infection, type, duration of course and are they effective, if patient tolerance of the catheter and associated drainage system, if the individual patient in any form of discomfort or pain…’ [Catheter Care: RCN guidance for nurses] |
| Social support (practical) | Advise on, arrange, or provide practical help (e.g. from friends, relatives, colleagues, ‘buddies’ or staff) for performance of the behaviour | ‘Organisations may like to make use of the tools developed by NICE to help implementation of the clinical guideline on Infection Control (Update). All the implementation tools can be found on the NICE website.’ [NICE Catheter audit Tool] |
| Information about health consequences | Provide information (e.g. written, verbal, visual) about health consequences of performing the behaviour. Note: consequences can be for any target, not just the recipient(s) of the intervention; | ‘Antibiotics are not effective for treating asymptomatic bacteriuria in adults with catheters or non-pregnant women. Unnecessary treatment with antibiotics can also increase the resistance of bacteria that cause urinary tract infections, making antibiotics less effective for future use.’ [NICE QS90: Urinary Tract Infections in Adults] |
| Information about emotional consequences | Provide information (e.g. written, verbal, visual) about emotional consequences of performing the behaviour. Note: consequences can be related to emotional health disorders (e.g. depression, anxiety) and/or states of mind (e.g. low mood, stress); consequences can be for any target, not just the recipient(s) of the intervention | ‘Where to perform a TWOC and why: at home, if possible, as it is more relaxed for the patient’ [Catheter Care: RCN guidance for nurses] |
| Information about social environmental consequences | Provide information (e.g. written, verbal, visual) about social and environmental consequences of performing the behaviour. Note: consequences can be for any target, not just the recipient(s) of the intervention; | **‘**The cost-effectiveness model developed for this guideline combined evidence of clinical effectiveness, costs and quality of life with respect to symptomatic urinary tract infection and associated complications. The results of the analysis showed that reusable non-coated catheters were the most cost-effective option for intermittent self-catheterisation.’ [NICE Catheter audit tool] |
| Instruction on how to perform the behaviour | Advise or agree on how to perform the behaviour (includes ‘Skills training’) | ‘Always remember that catheter straps should be used to secure the catheter tube to the leg to prevent trauma’ [Catheter Passport] |
| Social comparison | Draw attention to others’ performance to allow comparison with the person’s own performance | Harm report dashboard that compares the performance of different healthcare organisations on a number of metrics, including urinary tract infections rates. [Safety Thermometer] |
| Demonstration of the behaviour | Provide an observable sample of the performance of the behaviour, directly in person or indirectly e.g. via film, pictures, for the person to aspire to or imitate (includes ‘Modelling’). | ‘observe model/manikin being catheterised…observe catheterisation performed by others on actual patients’ [Catheter Care: RCN guidance for nurses] |
| Prompts/cues | Introduce or define environmental or social stimulus with the purpose of prompting or cueing the behaviour. The prompt or cue would normally occur at the time or place of performance. | ‘reminders to review the continuing use or prompt the removal of catheters’ [EPIC 3] |
| Behavioural practice/ rehearsal | Prompt practice or rehearsal of the performance of the behaviour one or more times in a context or at a time when the performance may not be necessary, in order to increase habit and skill. | ‘practise catheterisation on a model/manikin under supervision until confident’ [Catheter Care: RCN guidance for nurses] |
| Credible source | Present verbal or visual communication from a credible source in favour of or against the behaviour. Note: code this BCT if source generally agreed on as credible e.g., health professionals, celebrities or words used to indicate expertise or leader in field and if the communication has the aim of persuading. | ‘The tool was developed by a community of users who iteratively tested the tool to ensure it is quick and easy to use and gives useful information which can be used for local improvement’ [Safety Thermometer] |
| Identification of self as a role model | Inform that one's own behaviour may be an example to others. | ‘become a competent mentor for others’ [Catheter Care: RCN guidance for nurses] |
| Reward (outcome) | Arrange for the delivery of a reward if and only if there has been effort and/or progress in achieving the behavioural outcome (includes ‘Positive reinforcement’) Note: this includes social, material, self and non-specific rewards for outcome. | ‘Step 4: Working out the CQUIN payment’ [Safety Thermometer] |
| Restructuring the social environment | Change, or advise to change the social environment in order to facilitate performance of the wanted behaviour or create barriers to the unwanted behaviour (other than prompts/cues, rewards and punishments). | ‘This intervention allows nurses to remove catheters without asking for a physician written order’ [HOUDINI protocol] |
