# Supplementary material for: Reducing catheter-associated urinary tract infections: a systematic review of barriers and facilitators and strategic behavioural analysis of interventions
Source: Implement Sci. 2020 Jul 6;15:44. doi: 10.1186/s13012-020-01001-2 (PMC7336619; doi:10.1186/s13012-020-01001-2)
Supplement: Supplementary file 14 — Additional file 14. Opportunities for intervention design: the frequency with which theoretically congruent BCTs with important theoretical domains were used in existing interventions [file 13012_2020_1001_MOESM14_ESM.docx]

**Additional file 14. Opportunities for intervention design: the frequency with which theoretically congruent BCTs with important theoretical domains were used in existing interventions**

| **BCTs paired with domain as per predefined matrix (Additional file 6)** | **BCT Frequency, *n* interventions** | **% Potential relevant BCTs used at least once** |  |
| --- | --- | --- | --- |
| Memory, Attention, Decision Processes | | | |
| Self-monitoring of behaviour | 5 | 100% |  |
| Self-monitoring of outcome of behaviour | 3 |  |  |
| Action planning | 1 |  |  |
| Prompts and cues | 1 |  |  |
| Knowledge | | | |
| Information on health consequences | 9 | 57% |  |
| Biofeedback | 0 |  |  |
| Antecedents | 0 |  |  |
| Feedback on behaviour | 3 |  |  |
| Information on social/ environmental consequences | 4 |  |  |
| Information emotional consequences | 1 |  |  |
| Salience of consequences | 0 |  |  |
| Beliefs about consequences | | | |
| Information about emotional consequences | 1 | 50% |  |
| Salience of consequences | 0 |  |  |
| Covert Sensitization | 0 |  |  |
| Anticipated regret | 0 |  |  |
| Information about social/ environmental consequences | 4 |  |  |
| Pros and Cons | 0 |  |  |
| Vicarious reinforcement | 0 |  |  |
| Threat | 0 |  |  |
| Comparative imagining of future outcomes | 0 |  |  |
| Self-monitoring of behaviour | 5 |  |  |
| Self-monitoring of outcome of behaviour | 3 |  |  |
| Information on health consequences | 9 |  |  |
| Feedback on behaviour | 3 |  |  |
| Biofeedback | 0 |  |  |
| Feedback on outcome(s) of behaviour | 3 |  |  |
| Persuasive communication (Credible source) | 3 |  |  |
| Social Influences | | | |
| Social comparison | 1 | 50% |  |
| Social support (unspecified) | 0 |  |  |
| Social support (emotional) | 0 |  |  |
| Social support (practical) | 4 |  |  |
| Information about others’ approval | 0 |  |  |
| Vicarious consequences/ reinforcement | 0 |  |  |
| Restructuring the social environment | 1 |  |  |
| Identification of self as a role model | 1 |  |  |
| Social Reward | 0 |  |  |
| Demonstration of the behaviour | 2 |  |  |
| Social Professional Role and Identity | | | |
| Social support (unspecified) | 0 | 33% |  |
| Social support (emotional) | 0 |  |  |
| Social support (practical) | 4 |  |  |
| Environmental Context and Resources | | | |
| Restructuring the physical environment | 0 | 33% |  |
| Discriminative (learned) cue | 0 |  |  |
| Prompts/ Cues | 1 |  |  |
| Avoidance/ changing exposure to cues for the behaviour | 0 |  |  |
| Adding objects to the environment | 0 |  |  |
| Restructuring the social environment | 1 |  |  |
